# Impact of Intermittent Screening and Treatment for Malaria among School Children in Kenya: A Cluster Randomised Trial

**DOI:** 10.1371/journal.pmed.1001594

**Published:** 2014-01-28

**Authors:** Katherine E. Halliday, George Okello, Elizabeth L. Turner, Kiambo Njagi, Carlos Mcharo, Juddy Kengo, Elizabeth Allen, Margaret M. Dubeck, Matthew C. H. Jukes, Simon J. Brooker

**Affiliations:** 1 Faculty of Infectious and Tropical Diseases, London School of Hygiene & Tropical Medicine, London, United Kingdom; 2 Health Systems and Social Science Research Group, Kenya Medical Research Institute-Wellcome Trust Research Programme, Kilifi, Kenya; 3 Department of Biostatistics and Bioinformatics and Duke Global Health Institute, Duke University, Durham, North Carolina, United States of America; 4 Division of Malaria Control, Ministry of Public Health & Sanitation, Nairobi, Kenya; 5 Health and Literacy Intervention Project, Ukunda, Kenya; 6 Faculty of Epidemiology and Population Health, London School of Hygiene & Tropical Medicine, London, United Kingdom; 7 Department of Teacher Education, College of Charleston, South Carolina, United States of America; 8 Graduate School of Education, Harvard University, Cambridge, Massachusetts, United States of America; 9 Malaria Public Health Department, Kenya Medical Research Institute-Wellcome Trust Research Programme, Nairobi, Kenya; Mahidol-Oxford Tropical Medicine Research Unit, Thailand

## Abstract

**Background:**

Improving the health of school-aged children can yield substantial benefits for cognitive development and educational achievement. However, there is limited experimental evidence of the benefits of alternative school-based malaria interventions or how the impacts of interventions vary according to intensity of malaria transmission. We investigated the effect of intermittent screening and treatment (IST) for malaria on the health and education of school children in an area of low to moderate malaria transmission.

**Methods and Findings:**

A cluster randomised trial was implemented with 5,233 children in 101 government primary schools on the south coast of Kenya in 2010–2012. The intervention was delivered to children randomly selected from classes 1 and 5 who were followed up for 24 months. Once a school term, children were screened by public health workers using malaria rapid diagnostic tests (RDTs), and children (with or without malaria symptoms) found to be RDT-positive were treated with a six dose regimen of artemether-lumefantrine (AL). Given the nature of the intervention, the trial was not blinded. The primary outcomes were anaemia and sustained attention. Secondary outcomes were malaria parasitaemia and educational achievement. Data were analysed on an intention-to-treat basis.

During the intervention period, an average of 88.3% children in intervention schools were screened at each round, of whom 17.5% were RDT-positive. 80.3% of children in the control and 80.2% in the intervention group were followed-up at 24 months. No impact of the malaria IST intervention was observed for prevalence of anaemia at either 12 or 24 months (adjusted risk ratio [Adj.RR]: 1.03, 95% CI 0.93–1.13, *p* = 0.621 and Adj.RR: 1.00, 95% CI 0.90–1.11, *p* = 0.953) respectively, or on prevalence of *P. falciparum* infection or scores of classroom attention. No effect of IST was observed on educational achievement in the older class, but an apparent negative effect was seen on spelling scores in the younger class at 9 and 24 months and on arithmetic scores at 24 months.

**Conclusion:**

In this setting in Kenya, IST as implemented in this study is not effective in improving the health or education of school children. Possible reasons for the absence of an impact are the marked geographical heterogeneity in transmission, the rapid rate of reinfection following AL treatment, the variable reliability of RDTs, and the relative contribution of malaria to the aetiology of anaemia in this setting.

**Trial registration:**

www.ClinicalTrials.gov NCT00878007

*Please see later in the article for the Editors' Summary*

## Abstract 2

Katherine Halliday and colleagues conducted a cluster randomized controlled trial in Kenyan school children in an area of low to moderate malaria transmission to investigate the effect of intermittent screening and treatment of malaria on health and education.

*Please see later in the article for the Editors' Summary*

## Editors' Summary

**Background:**

Every year, more than 200 million cases of malaria occur worldwide and more than 600,000 people, mostly children living in sub-Saharan Africa, die from this mosquito-borne parasitic infection. Malaria can be prevented by controlling the night-biting mosquitoes that transmit *Plasmodium* parasites and by sleeping under insecticide-treated nets to avoid mosquito bites. Infection with malaria parasites causes recurring flu-like symptoms and needs to be treated promptly with antimalarial drugs to prevent the development of anaemia (a reduction in red blood cell numbers) and potentially fatal damage to the brain and other organs. Treatment also reduces malaria transmission. In 1998, the World Health Organization and several other international bodies established the Roll Back Malaria Partnership to provide a coordinated global approach to fighting malaria. In 2008, the Partnership launched its Global Malaria Action Plan, which aims to control malaria to reduce the current burden, to eliminate malaria over time country by country, and, ultimately, to eradicate malaria.

**Why Was This Study Done?:**

In recent years, many malaria-endemic countries (countries where malaria is always present) have implemented successful malaria control programs and reduced malaria transmission levels. In these countries, immunity to malaria is now acquired more slowly than in the past, the burden of clinical malaria is shifting from very young children to older children, and infection rates with malaria parasites are now highest among school-aged children. Chronic untreated *Plasmodium* infection, even when it does not cause symptoms, can negatively affect children's health, cognitive development (the acquisition of thinking skills), and educational achievement. However, little is known about how school-based malaria interventions affect the health of children or their educational outcomes. In this cluster randomized trial, the researchers investigate the effect of intermittent screening and treatment (IST) of malaria on the health and education of school children in a rural area of southern Kenya with low-to-moderate malaria transmission. Cluster randomized trials compare the outcomes of groups (“clusters”) of people randomly assigned to receive alternative interventions. IST of malaria involves periodical screening of individuals for *Plasmodium* infection followed by treatment of everyone who is infected, including people without symptoms, with antimalarial drugs.

**What Did the Researchers Do and Find?:**

The researchers enrolled more than 5,000 children aged between 5 and 20 years from 101 government primary schools in Kenya into their 24-month study. Half the schools were randomly selected to receive the IST intervention (screening once a school term for infection with a malaria parasite with a rapid diagnostic test [RDT] and treatment of all RDT-positive children, with or without malaria symptoms, with six doses of artemether-lumefantrine), which was delivered to randomly selected children from classes 1 and 5 (which contained younger and older children, respectively). During the study, 17.5% of the children in the intervention schools were RDT-positive at screening on average. The prevalences of anaemia and parasitemia (the proportion of children with anaemia and the proportion who were RDT-positive, respectively) were similar in the intervention and control groups at the 12-month and 24-month follow-up and there was no difference between the two groups in classroom attention scores at the 9-month and 24-month follow-up. The IST intervention also had no effect on educational achievement in the older class but, unexpectedly, appeared to have a negative effect on spelling and arithmetic scores in the younger class.

**What Do These Findings Mean?:**

These findings indicate that, in this setting in Kenya, IST as implemented in this study provided no health or education benefits to school children. The finding that the educational achievement of younger children was lower in the intervention group than in the control group may be a chance finding or may indicate that apprehension about the finger prick needed to take blood for the RDT had a negative effect on the performance of younger children during educational tests. The researchers suggest that their failure to demonstrate that the school-based IST intervention they tested had any long-lasting health or education benefits may be because, in a low-to-moderate malaria transmission setting, most of the children screened did not require treatment and those who did lived in focal high transmission regions, where rapid re-infection occurred between screening rounds. Importantly, however, these findings suggest that school screening using RDT could be an efficient way to identify transmission hotspots in communities that should be targeted for malaria control interventions.

**Additional Information:**

Please access these websites via the online version of this summary at http://dx.doi.org/10.1371/journal.pmed.1001594.

- This study is further discussed in a *PLOS Medicine* Perspective by Lorenz von Seidlein
- Information is available fro m the World Health Organization on malaria (in several languages); the 2012 World Malaria Report provides details of the current global malaria situation
- The US Centers for Disease Control and Prevention provide information on malaria (in English and Spanish), including a selection of personal stories about children with malaria
- Information is available from the Roll Back Malaria Partnership on the global control of malaria and on the Global Malaria Action Plan (in English and French); its website includes a fact sheet about malaria in Kenya
- MedlinePlus provides links to additional information on malaria (in English and Spanish)
- More information about this trial is available
- More information about malaria control in schools is provided in the toolkit

## Introduction

In many malaria endemic countries, successful control programmes have recently reduced the level of malaria transmission [1]–[3], and as a consequence, immunity to malaria is acquired more slowly and the burden of clinical malaria is shifting from the very young to older children [4],[5]. Recent success in malaria control has also prompted a renewed emphasis on malaria elimination, leading to a shift in focus from targeting only clinical malaria to also identifying and treating asymptomatic malaria parasitaemia [1],[6]. Infection rates are typically highest among school-aged children [7],[8], who, due to recent improvements in primary school access, are increasingly enrolled in school [9],[10]. Tackling such parasitaemia, whether or not it results in clinical disease, is important for two reasons. First, an increasing body of evidence is showing that chronic untreated *Plasmodium* infections can negatively affect children's health [11],[12] and cognitive function [13]–[15], including sustained attention [16], and ultimately, their educational achievement [17],[18]. Second, with the move towards elimination in low-moderate transmission settings [19],[20], there is a need to tackle untreated reservoirs of infection, to which school children are important contributors [21],[22]. Yet, surprisingly, there remains a lack of consistent policy and technical guidance [23] on which interventions can reduce the burden of malaria among school children and which can cost-effectively be delivered through existing school systems.

Previous studies have highlighted the beneficial impact of school-based intermittent preventive treatment (IPT) on health and cognitive function in high [24] and high, seasonal [25] malaria transmission settings. However, the recent withdrawal of the primary drugs for IPT, sulphadoxine-pyrimethamine (SP) and amodiaquine (AQ), in many east African countries, precluded further investigation of IPT using SP+AQ. A possible alternative to IPT is intermittent screening and treatment (IST), whereby individuals are periodically screened for *Plasmodium* infection using a rapid diagnostic test (RDT) and those infected (whether symptomatic or not) are treated with a full course of first-line drug treatment, artemether-lumefantrine (AL). The potential of IST was first highlighted by modelling work [26],[27], and its comparable efficacy to IPT in antenatal care [28] has been evaluated, although a recent trial in Burkina Faso indicated no impact of IST on community-wide malaria transmission [29]. This paper reports the results of a cluster randomised trial investigating the impact of IST in schools on health and education outcomes in school children in a low-moderate transmission setting on the south coast of Kenya [30].

## Methods

The original protocol for the trial (Protocol S1) and the supporting CONSORT checklist (Checklist S1) are provided as supporting information. Trial instruments and data are available on the World Bank Microdata catalogue at: http://microdata.worldbank.org/index.php/catalog/671.

### Ethics Statement

The study was approved by the Kenya Medical Research Institute and National Ethics Review Committee (SSC number 1543), the London School of Hygiene & Tropical Medicine Ethics Committee (5503), and the Harvard University Committee on the Use of Human Subjects in Research (F17578-101). Prior to the randomisation, meetings were held with community and school leaders and parents/guardians in each school to explain the study objectives and procedures. Parents/guardians of all children in classes 1 and 5 were requested to provide individual written informed consent and they were given the option to withdraw their child from the study at any time. Prior to every IST round or assessment, the procedures were explained to the children and they were required to provide verbal assent. An independent data monitoring committee reviewed the trial protocol, data analysis plan, and preliminary results.

### Study Area and Population

**Figure 1:**
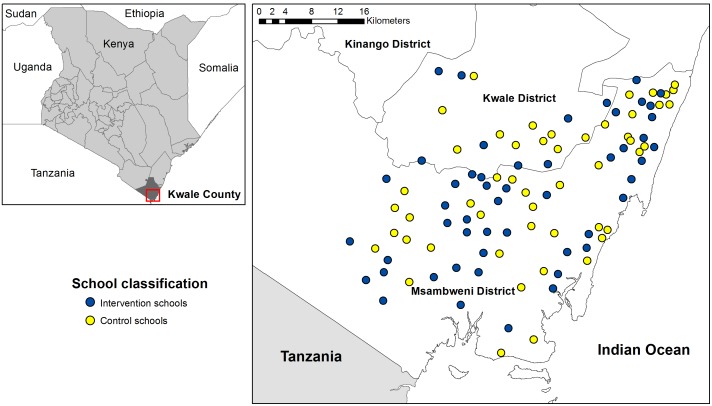
Map of the study area and schools. Schools assigned to the IST intervention are shown in blue and schools assigned to the control group are shown in yellow. Insert shows the location of the study site in Kenya.

The trial was conducted from January 2010 to March 2012 in Kwale and Msambweni districts on the south Kenyan coast (Figure 1). Malaria transmission in the area is moderate and perennial with seasonal peaks following the two rainy seasons (April–July and September–November) [31]. The primary malaria vectors are *Anopheles gambiae s.l.* and *A. funestus* [32],[33]. Intensity of malaria transmission has been declining in recent years: school surveys conducted in 2010 reported prevalences of *P. falciparum* of 9%–24% [34],[35], compared to 64% in 1998 [32]. Overall reported net use in the region is high, with the communities having benefited from universal coverage campaigns. During the two-year trial period, albendazole was delivered through households as part of the national lymphatic filariasis campaign in 2011, although coverage was not extensive and praziquantel was delivered to schools in the area in June 2011. The vast majority of the population in these districts belong to the Mijikenda ethnic group, with Digo and Duruma the predominant subgroups [36]. The region is primarily rural with subsistence farming of maize and cassava practiced by many of the communities, although titanium mining has recently become an important source of employment. In economic and educational terms, the districts are ranked the seventh poorest in Kenya and consistently have some of the worst performing schools in the national school examinations [37].

Kwale District has 85 schools across four zones, and in two of these an alternative literacy intervention study was underway. Therefore only 20 schools from Mkongani and Shimba Hills zones were included in our study, allowing the two interventions to proceed without leakage. In Msambweni District 81 of the 112 schools were selected, with schools in Lunga Lunga and Mwereni zones greater than 70 km away from the project office excluded because of logistical considerations in visiting them.

### Study Design

**Figure 2:**
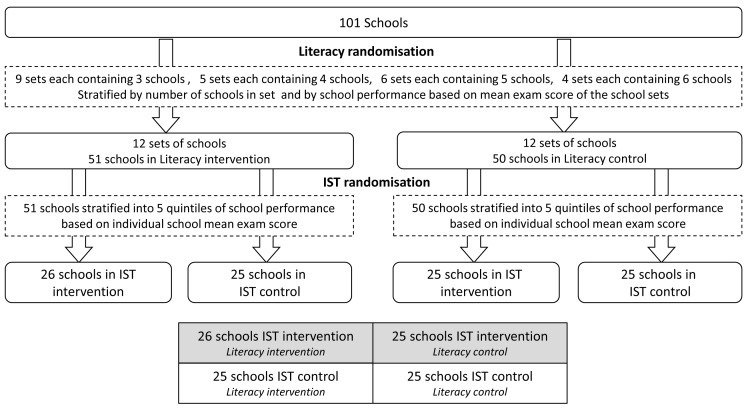
Study design diagram. This figure depicts the randomisation procedures.

The study was designed as a factorial cluster randomised trial to investigate the impact of two interventions: (i) the impact of school-based IST for malaria on the health, sustained attention, and education of school children, and (ii) the impact of a literacy intervention on education. In order to evaluate the potential interaction between the two interventions, schools were randomised to one of four groups, receiving either: (i) IST alone; (ii) the literacy intervention alone; (iii) both interventions combined; or (iv) control group where neither intervention was implemented (Figure 2). The study was not blinded. Because of the factorial design of the trial and lack of interaction detected (interaction effect *p*-values of 0.45, 0.26, and 0.60 for the three key literacy outcomes) between the two interventions in class 1 where both were implemented, we report the results of the interventions separately. Only the IST intervention results are reported in this paper. The results of the literacy intervention will be reported in a separate paper targeting an education research audience as the literacy intervention was focused purely on enhanced English and Swahili literacy instruction and was not intended to have an impact on health.

Recruitment and baseline sample collection were conducted in January–March 2010 using children randomly selected from classes 1 (age range: 5–15 years) and 5 (age range: 8–20 years). Both classes received the IST intervention, but the literacy intervention was targeted only to children in class 1 and as they advanced to class 2, as it focused on the initial stages of literacy acquisition. Education outcome measures were assessed in the same children at 9 and 24 months and health outcome measures at 12 and 24 months. Full details of the eligibility, randomisation, intervention procedures, and baseline results have been presented elsewhere [30],[35]. The study is registered with ClinicalTrials.gov, NCT00878007.

### Sample Size

The sample size was based on methods designed for cluster randomised trials and assumed that 101 eligible schools would be randomised to the four intervention groups, with an average of 50 children per school. On the basis of data collected previously in the study area, the baseline prevalence of anaemia was assumed to be 20% and the coefficient of variation (CV) 0.2 [30]. In order to detect a 25% reduction in the prevalence of anaemia between the two groups, based on previous work in Kenya [24], the sample size required to give a study with a power of 80% at a two-sided significance level of 5%, was a total of 27 schools in each arm with 50 children per school. A sample size of 101 schools with 25 children per class (i.e., analysing classes 1 and 5 separately) will enable us to detect, with 80% power and 5% significance, an approximate difference of 0.2 standard deviations (SDs) between arms of the trial in educational achievement (assuming an intraclass correlation coefficient [ICC] of 0.2 and a pre-post correlation of 0.7), and a difference of approximately 0.15 SD in tests of sustained attention (assuming an ICC of 0.1 and a pre post correlation of 0.7) [24]. The increased number of schools required for the sustained attention and educational achievement outcomes provided greater power (97%) to detect a 25% reduction in the prevalence of anaemia, or alternatively 85% power to detect a 20% reduction.

### Randomisation

The 101 schools were randomised in two stages (Figure 2), with each stage conducted during a public ceremony. In Kenya, schools are aggregated into sets of between three and six closely located schools, which regularly meet and share information, supported by a Ministry of Education Teacher Advisory Centre tutor. Our 101 study schools formed 24 of these sets of schools, which were randomised either to receive the literacy intervention or to serve as the literacy control. Randomisation of these sets of schools was stratified by (i) set size, to ensure equal numbers of schools in the experimental groups, and (ii) average primary school leaving exam scores of the school sets, to balance the two study groups for school achievement. This randomisation procedure was designed to minimize contamination of the literacy intervention methods across the study groups. In stage two, the IST intervention was randomly allocated at the level of the school, with the 101 schools re-stratified by (i) literacy intervention group assignment and (ii) quintiles of average school exam scores, producing ten strata overall.

### Enrolment

At enrolment, children's height and weight were measured, axillary temperature was digitally recorded, and finger-prick blood samples were obtained to prepare thin and thick blood films and to determine haemoglobin concentration (Hb). Children known or suspected to be homozygous for sickle cell trait or pregnant were excluded. Any child found with Hb<80 g/l was referred by the nurse to the nearest health facility for iron therapy, and any child found with Hb<50 g/l was taken to the hospital for transfusion. Baseline parasitaemia was measured in the intervention group during the first round of screening but was not measured in the control group owing to the ethical constraints of testing for malaria but not treating children found to be infected in the control schools, which was of particular importance at baseline as the intervention involved screening of *Plasmodium* infection.

A questionnaire was administered to parents/guardians to record information on residence, family size, ownership of possessions, mosquito net use by them and their children, recent deworming of the child, house construction, and parental education level.

### Intervention

IST was outlined as a possible strategy in the “*Malaria-Free Schools Initiative*,” as part of the Kenya National Malaria Strategy 2009–2017 [38]. During IST, children were screened once a school term for malaria parasitaemia using an RDT (ParaCheck-*Pf* device, Orchid Biomedical Systems), which is able to detect *P. falciparum*. Screening was conducted by laboratory technicians. Repeat visits were made to follow-up children absent on the day of screening. Children (with or without malaria symptoms) found to be RDT-positive were treated with a six dose regimen of AL (artemether 20 mg/lumefantrine 120 mg, Coartem, Novartis) over three days. Doses of AL were based on weight, with children stratified into one of the four categories (<15 kg, 15–24.9 kg, 25–34.9 kg, and ≥35 kg). AL was given at a dose of 20/120 mg to children <15 kg, 40/240 mg to children 15–24.9 kg, 60/360 mg to children 25–34.9 kg, and 80/480 mg to those who weighed ≥35 kg. Parents or older siblings of children were called and a nurse explained that their child was infected with malaria parasites and required treatment. Doses 1, 3, and 5 were given under direct observation at the school by the study nurses. Children were given milk and biscuits with the AL and observed for 30 minutes after drug administration. If vomiting occurred during this period, drugs were re-administered. If vomiting occurred on a second occasion, this was noted but the drugs were not given again. Such children were not excluded from the trial and they were eligible to receive drugs on the subsequent two days. The parents/older siblings or study children themselves if in the older classes were given doses 2, 4, and 6 each day for evening administration and provided with instructions on treatment. Children absent from school on days two or three of treatment were followed up at their home by the nurse, and provided with the doses. Supervised treatment was defined as nurses administering and directly observing doses 1, 3, and 5 taken on three consecutive mornings in the school and recording doses 2 and 4 reported by the child as having been taken the previous evenings. No direct confirmation of whether dose 6 was taken was recorded by the nurse. The record of supervised treatment was used as a proxy for compliance. Five rounds of screening and treatment were implemented. The first round was conducted alongside baseline health assessments in March 2010, the second round in July 2010, the third in September 2010, the fourth in March 2011, and the final round in October 2011.

Adverse events were monitored by the study team for 24 hours after each treatment, and a further 28 days thereafter using a passive surveillance system in schools. Travel costs were reimbursed and treatment charges waived. Adverse experiences were monitored until the event was cured or had stabilised. Agranulocytosis and hepatotoxicity were not assessed because of logistical constraints.

### Follow-up

Cross sectional health surveys were carried out at 12 and 24 months. During these surveys, temperature, weight, and height were measured and a finger-prick blood sample was collected for determination of malaria parasitaemia and Hb. Children with an axillary temperature ≥37.5°C were tested using an RDT, providing an on-the-spot diagnosis for malaria, and treatment was administered as per national guidelines.

### Laboratory Methods

Hb was measured using a portable haemoglobinometer (Hemocue). Thick and thin blood films were stained with Giemsa, asexual parasites were counted against 200 white blood cells (WBCs), and parasite density was estimated assuming an average WBC count of 8,000 cells/µl. A smear was considered negative after reviewing 100 high-powered fields. Thin blood smears were reviewed for species identification. All blood slides were read independently by two microscopists who were blinded to group allocation. Discrepant results were resolved by a third microscopist.

### Attention and Educational Achievement

Tests of sustained attention and educational achievement were administered at baseline, 9 months, and 24 months. Sustained attention was a primary outcome, assessed through the code transmission test, adapted from the TEA-Ch (Tests of everyday attention for children) battery [39]. A recorded list of digits is read aloud and children are required to listen for a code—two consecutive occurrences of the number 5—and then record the number(s) that preceded the code. To avoid floor effects, (in which the assessment is too challenging to establish the range of abilities in the target population), a simpler measure of sustained attention—the pencil tap test [40] —was used at baseline for the younger cohort. Children were required to tap a pencil on the desk a predetermined number of times in response to the assessor's taps. The secondary outcome of educational achievement was measured through tests of literacy and numeracy. Literacy was assessed through group administered English spelling tests, adapted from PALS (Phonological Awareness Literacy Screening) [41], with the younger classes asked to spell five three-letter words and credit given for phonetically acceptable choices for each letter and the older classes asked to spell 25 words with credit given for correctly spelling the features and sound combinations of the word. Numeracy assessments involved an oral test of basic arithmetic for younger children at baseline and 9-month follow-up and written arithmetic at 24-month follow-up and a written arithmetic test throughout for older children. All educational assessments were piloted prior to use in the baseline and follow-up evaluations. During piloting the assessments were conducted under the same assessment conditions on two occasions a week apart, with the correlation between the scores at the two time points providing a reliability score. The inclusion criteria for the tests used in this trial was a Cronbach's alpha correlation of 0.7 or above, indicating a well constructed test with consistent administration.

The educational assessments were conducted separately to the health assessment both for logistical reasons and so as not to cause bias during the educational assessments due to apprehension of the finger-prick. The education assessments preceded the latter by an average of a week at baseline and 24-month follow-up. However, during the first follow-up, the education assessments were conducted at the end of the school year (9 months) and the health assessments were conducted at end of a full year (12 months).

### Data Analysis

Data were double-entered, consistency checks were performed, and all analysis was conducted using Stata software version 12.1. The pre-specified primary outcome measures were the prevalence of anaemia, defined according to age and sex corrected World Health Organization (WHO) thresholds: Hb<110 g/l in children under 5 years; <115 g/l in children 5 to 11 years; <120 g/l in females 12 years and over and males 12 to 15 years old; and <130 g/l in males over 15 years, with no adjustment made for altitude [42] and sustained attention. The pre-specified secondary outcomes were the prevalence of *P. falciparum* and scores for spelling and arithmetic. Reported information on ownership of household assets and household construction was used to construct wealth indices using principal component analysis [43] and resulting scores were divided into quintiles. Anthropometric measurements were processed using the WHO Anthroplus Stata macro [44] to derive indicators of stunting, thinness, and underweight.

The analyses described here correspond to a pre-specified statistical analysis plan, approved by both the data monitoring committee and trial steering committee before any data were examined.

Baseline school and child characteristics, together with baseline measurements of the study outcomes, were summarized by study groups separately, with class-specific study outcomes reported separately by class. Counts and percentages were used for categorical variables. Means and standard deviations, or medians and the limits of the inter-quartile range (IQR), were reported for continuous variables. Coefficients of variation (CVs) for the binary (health) outcomes and intraclass correlation coefficients (ICCs) for the continuous (cognitive and education) outcomes were calculated from the baseline measures using appropriate formulae [45].

The effectiveness of the IST intervention was assessed using generalized estimating equations (GEE) with robust standard errors and an exchangeable correlation matrix to allow for clustering within schools. All main analyses used the intention-to-treat principle whereby children were analyzed in the intervention group that they were assigned to, even if the child moved schools or did not fully comply. The primary pre-specified analysis adjusted for age (as a continuous variable), sex, and the baseline measure of the outcome, except for baseline *P. falciparum*, which was not measured in the control schools. As randomisation of schools to the IST intervention was stratified on the basis of both literacy intervention assignment and school mean exam score (Figure 2), all adjusted analyses presented account for these two stratification factors. Data for classes 1 and 5 combined were used for the health outcome analyses. However, as different assessments were administered for classes 1 and 5 for the evaluation of attention (e.g., pencil tap for class 1 and code transmission for class 5), literacy, and numeracy outcomes, analyses were conducted for each class separately. Separate GEE analyses were conducted for the first and second follow-ups. No formal adjustment was made for multiple testing, therefore *p*-values should be interpreted with due caution. However, as specified in the statistical analysis plan, formal testing was restricted to two primary and three secondary pre-specified outcomes.

For comparison purposes, we also obtained estimates from an unadjusted model that did not adjust for baseline outcome measures, child characteristics, or study design (literacy group and mean school-exam score) and hence retained all study children assessed at follow-up regardless of whether they had baseline measures. Secondary analyses were conducted additionally adjusting for stunting, school-feeding programme, and socioeconomic status (SES) on top of the pre-specified variables. These additional adjustments had no notable impact on the effect estimates and are not presented.

In order to gain power and account for missing data, random effects models, using a likelihood-based approach, were fitted to the one-year and two-year follow-up data simultaneously (Tables S1, S2, S3, S4, S5, S6, S7; Text S1). Additional sensitivity analyses were conducted to examine intervention effects when children who had transferred from their original school were excluded from the analyses (Table S8).

### Role of the Funding Source

The funders had no role in the study design, data collection, data analysis, data interpretation, or writing of the report. The corresponding author had full access to all the data in the study and had final responsibility for the decision to submit for publication.

## Results

### Trial Profile and Baseline Data

**Figure 3:**
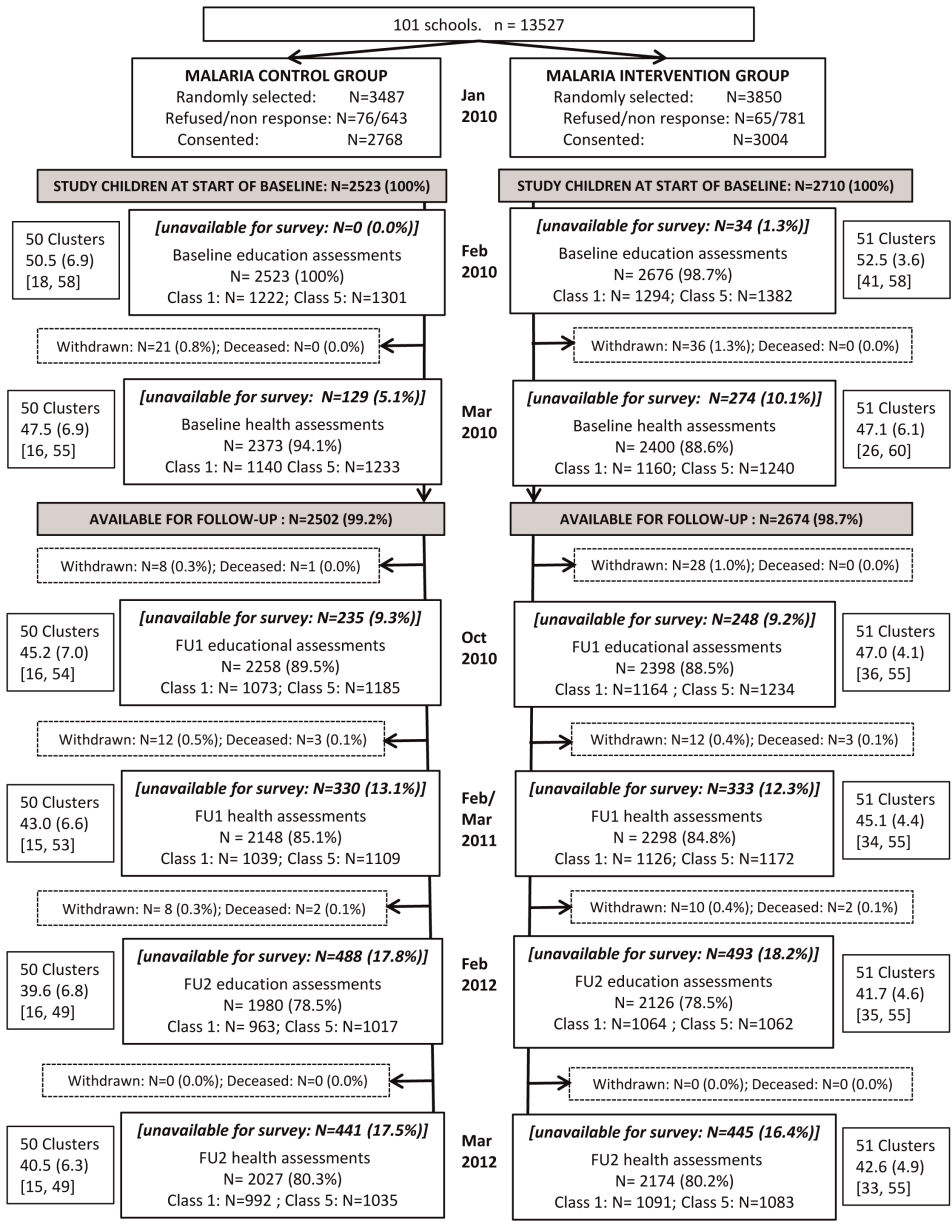
Trial profile. The flow of children and clusters in the 50 control 51 IST intervention groups at all assessment points throughout the two-year study period. FU1 indicates follow-up 1 and FU2 indicates follow-up 2. Cluster size is presented as mean (SD) [min, max].

One hundred and one schools were randomised to one of the two study groups (Figure 3). In total, 7,337 children aged between 5 and 20 years (median: 10 years and IQR: 8–13 years) were randomly selected in January 2010 of which 5,772 (78.7%) parents consented, with no real differences found between groups in terms of percentage of parents refusing and not attending the meetings. Overall, 5,233 children were initially enrolled, of which 5,176 (98.9%) children were eligible for follow-up after the baseline assessments. Characteristics of the children included in each of the study groups are shown in Table 1. The numbers of children per school ranged from 18 to 58 but overall were well balanced between groups (control: median, 52; IQR, 50–54 and intervention: median, 53; IQR, 50–55). A difference in percentage of children unavailable for the baseline health surveys was observed between the groups with 5.1% and 10.1% unavailable in the control and intervention groups, respectively (Figure 3).

**Table 1:** Baseline characteristics of 5,233 study children in the 50 control and 51 IST intervention schools.

Characteristics; *n* (%)^a^	Measure/Subcharacteristic	Control	Intervention
**School characteristics** b		**50 schools**	**51 schools**
Exam score	Mean (SD)	223.4 (27.7)	225.8 (29.0)
School size	Median (IQR) [min, max]	505 (308, 961) [85, 4,891]	568 (389, 692) [225, 1,344]
Enrolled class 1	Mean (SD) [min, max]	24.4 (3.3) [10],[30]	25.8 (1.5) [23],[30]
Enrolled class 5	Mean (SD) [min, max]	26.0 (4.6) [8],[30]	27.3 (3.3) [16],[32]
School programmes	Feeding	22 (44.0)	27 (52.9)
	De-worming	50 (100.0)	49 (96.1)
	Malaria control	9 (18.4)	12 (23.5)
**Child characteristics** b		**2,523 children**	**2,710 children**
Age^c^	Mean (SD)	10.1 (2.8)	10.3 (2.8)
	5–9	1,041 (41.2)	1,069 (39.5)
	10–12	877 (34.8)	925 (34.1)
	13–20	605 (24.0)	716 (26.4)
Sex	Male	1,257 (49.8)	1,319 (48.7)
Child sleeps under net	Usually	1,668 (67.3)	1,682 (63.1)
	Treated net^d^	1,357 (83.3)	1,308 (80.5)
	Last night^d^	1,606 (96.3)	1,609 (95.7)
Nutritional status	Underweight	266 (27.0)	231 (23.9)
	Stunted	600 (25.2)	612 (24.9)
	Thin	482 (20.2)	450 (18.3)
**Household characteristics** b			
Parental education	No schooling	726 (29.4)	925 (34.7)
	Primary schooling	1,292 (52.2)	1,381 (51.8)
	Secondary schooling	353 (14.3)	278 (10.4)
	Higher education	102 (4.1)	83 (3.1)
SES	Poorest	440 (17.7)	655 (24.4)
	Poor	483 (19.5)	564 (21.0)
	Median	465 (18.7)	495 (18.5)
	Less poor	524 (21.1)	509 (19.0)
	Least poor	572 (23.0)	458 (17.1)
Household size	1–5	697 (28.1)	703 (26.4)
	6–9	1,444 (58.3)	1,580 (59.3)
	10–31	338 (13.6)	382 (14.3)
**Study endpoints-baseline** e		**2,523 children**	**2,710 children**
**Anaemia prevalence** f **(k = 0.21)**	Age-sex specific	1,073 (45.2)	1,114 (45.5)
	Severe (<70 g/l)	14 (0.6)	14 (0.6)
	Moderate (70–89 g/l)	43 (1.8)	55 (2.2)
	Mild (90–109 g/l)	530 (22.3)	518 (21.1)
	None (≥110 g/l)	1,786 (75.3)	1,864 (76.1)
**Haemoglobin (g/l)**	Mean (SD)	117.3 (13.0)	117.5 (13.7)
***P. falciparum*** ** prevalence** f **^,^** g **(k = 1.03)**		—	311 (12.9)
**Class 1** f **^,^** h		**1,222 children**	**1,317 children**
Score: 0–20 (ICC = 0.07)	Sustained attention^i^	11.9 (6.7) [0, 20]	12.1 (6.6) [0, 20]
Score: 0–20 (ICC = 0.29)	Spelling	8.6 (4.5) [0, 19]	7.7 (4.4) [0, 20]
Score: 0–30 (ICC = 0.11)	Arithmetic	2.6 (2.4) [0, 17]	2.6 (2.5) [0, 15]
**Class 5** f **^,^** h		**1,301 children**	**1,393 children**
Score: 0–20 (ICC = 0.23)	Sustained attention^i^	9.9 (6.0) [0, 20]	10.4 (5.7) [0, 20]
Score: 0–78 (ICC = 0.09)	Spelling	27.9 (11.8) [0, 63]	25.8 (11.2) [1],[59]
Score: 0–38 (ICC = 0.22)	Arithmetic	29.4 (5.6) [0, 38]	28.5 (5.8) [0, 38]

a Percent of non-missing children in each study group presented for categorised data. For continuous data mean (SD) [min,max] is presented.

b All characteristics have less than 2% missing data with the exception of following indicators (reported as control/intervention): stunted and thin both (138/248 [5.5/9.2%] missing), underweight (1,538/1,744 [61.0/64.4%] missing), net use last night (661/840 [26.2/31.0%] missing).

c In Class 1, mean (SD) for age is: 7.8 (1.7) and in Class 5, mean (SD) for age is:12.5 (1.6).

d Percentages of treated nets and children sleeping under a net last night are presented only for those children who were reported as usually sleeping under a net.

e Study endpoints have less than 5% missing data at baseline with the exception of the following (reported as control/intervention): Hb (147/255 [5.8/9.4%] missing), *P. falciparum* infection (274 [10.1%] missing in intervention group), class 5 attention (79/72 [6.1/5.2%] missing).

f Coefficient of variation (k) estimated for binary outcomes using available baseline (i.e., only using data from IST schools for *P. falciparum*) and interclass correlation coefficient (ICC) estimated for continuous outcomes using baseline measures.

g Not measured at baseline in the control group.

h Presented as mean (SD) [min,max].

i In class 1 sustained attention was measured by the “pencil tap test” and in class 5 sustained attention was measured by the “two digit code transmission test.”

### Compliance with Screening and Treatment

During the 24 months of intervention, an average of 2,340 children (88.4% of eligible study children) in the 51 intervention schools were screened at each visit, of whom, on average 17.5% were RDT-positive (Table 2). Of the study children, 84.0% were screened at four or more IST rounds and 66.8% were screened at all five rounds. By the fifth screening round, 3.3% children were lost due to withdrawal or death and a further 17.7% of children were lost due to out-migration. The percentage of children RDT-positive at each screening ranged from 14.8% to 19.2%, with no distinct trend over time. Overall, 99.1% of RDT-positive results led to treatment across the five screening rounds and 92.6% of these were recorded as receiving the fully supervised six-dose treatment regime (Table 2). There was an apparent decline in full supervision (a proxy for compliance) with time, falling from 96.9% at the first round to 81.7% at the fifth round. RDT performance, examined against a “gold standard” of expert microscopy, revealed consistently high specificity, greater than 90% at all rounds, whereas sensitivity was more variable ranging from 68.7% to 94.6% across surveys, with higher sensitivity observed during the wet season compared to the dry season (Table 2).

**Table 2:** Summary information for 2,710 study children in the IST intervention group by screening round.

IST Round	Season	Study Children^a^	*n* (%) Screened	*n* (%) RDT Positive	*n* (%) Treated	*n* (%) Supervised Treatment^b^	RDT Sensitivity/Specificity^c^
Feb–Mar 2010	Dry	2,674 (98.7)	2,454 (91.8)	453 (18.5)	449 (99.1)	435 (96.9)	78.5/90.6
Jun–Jul 2010	Wet	2,654 (97.9)	2,430 (91.6)	466 (19.2)	465 (99.8)	440 (94.6)	89.2/90.4
Sept 2010	Wet	2,651 (97.8)	2,368 (89.3)	444 (18.8)	443 (99.8)	422 (95.3)	94.6/90.3
Feb–Mar 2011	Dry	2,631 (97.1)	2,291 (87.1)	340 (14.8)	335 (98.5)	306 (91.3)	68.7/91.9
Oct 2011	Wet	2,621 (96.7)	2,157 (82.3)	345 (16.0)	338 (98.0)	276 (81.7)	NA
**TOTALS**		**13,231**	**11,700 (88.4)**	**2,048 (17.5)**	**2,030 (99.1)**	**1,879 (92.6)**	**82.7/90.8**

Sensitivity and specificity of RDTs compared to expert microscopy is displayed.

a Study children are shown as a percentage of the 2,710 initially eligible for the intervention and loss at each stage represents withdrawals and/or deaths. Child transfer events are not included.

b Children treated who were directly observed taking doses 1, 3, and 5 in school at the correct time and who reported taking the evening doses.

c Microscopy results not available for visit 5.

### Follow-up

Of the 5,233 children enrolled initially, 4,446 (85.0%) were included in the 12-month follow-up health survey and 4,201 (80.3%) were included in the 24-month health survey (Figure 3). At 12 and 24 months, children lost to follow-up across both study arms were largely similar to children followed up (Tables S1 and S2), with slightly lower spelling scores in those children lost to follow-up across both groups and a higher proportion of children whose parents had no schooling in those lost to follow-up in the intervention schools. The prevalence of *P. falciparum*, in the intervention group, was lower in children lost to follow-up (8.6%) compared to those followed-up (13.6%) at both 12 and 24 months.

Overall, 4,656 (89.0%) of children were included in the 9-month follow-up education survey and 4,106 (78.5%) in the 24-month follow-up survey. Children unavailable for the follow-up educational surveys at 9 and 24 months were similar across the two study groups (Tables S4 and S5), with a slight imbalance in SES and parental education categories seen between children available and unavailable for the survey in the intervention group. Additionally, baseline prevalence of *P. falciparum* was lower in children lost to follow-up (9.1%) compared to those followed-up (13.3%) in the intervention arm.

As intention-to-treat analysis was performed, no adjustment was made for children transferring between schools and study groups at the follow-ups. Overall, 308 children were recorded as transferred by the end of the study. Of those, 46 (0.9%), 71 (1.8%), and 308 (5.9%) children were assessed in a different school from their initial enrolment school, at 9-month, 12-month, and 24-month follow-ups, respectively. Sensitivity analysis excluding these transfers resulted in no change in direction or magnitude of results (Table S8).

### Effect of IST on Anaemia and *P. falciparum* Infection

At 12-months follow-up, 2,148 children in the control schools and 2,298 in the intervention schools provided a finger-prick blood sample for Hb assessment, and at 24 months 2,027 and 2,174 children provided finger-prick samples in the control and intervention groups, respectively. There was no significant difference in the prevalence of anaemia between children in the two groups at 12- or 24-month follow-ups (adjusted risk ratio [Adj.RR]: 1.03, 95% CI 0.93–1.13, *p* = 0.621 and Adj.RR: 1.00, 95% CI 0.90–1.11, *p* = 0.953), respectively (Table 3); the same was observed in relation to mean Hb. There was also no significant difference in the prevalence of *P. falciparum* between study groups at 12 or 24 months. Subgroup analysis of the impact of IST intervention on anaemia according to *Plasmodium* infection prevalence at baseline (using 12-month estimates for the control group as a proxy for baseline), demonstrated no differential impact by prevalence category (<5%, 5%–19%, and 20%+) at either follow-up. Similarly, no difference was seen when analysis was stratified, within the intervention group only, by numbers of treatments received across the study period (Tables S9 and S10).

**Table 3:** Effect of the IST intervention at 12- and 24-months follow-up on health outcomes anaemia and *P. falciparum* prevalence for study children.

Outcome	Control (50 Schools)		Intervention (51 Schools)		Risk Ratio^a^ (95% CI)	*p*-Value	Cluster-Size; Range (Average)
	*N*	*n* (%)^b^	*N*	*n* (%)^b^			
**12-MONTHS FOLLOW-UP**	2,478		2,631				
**Prevalence of anaemia** c							
Unadjusted	2,146	837 (39.0%)	2,297	920 (40.1%)	1.03 (0.91,1.16)	0.646	15–55 (44.0)
Adjusted	2,048	788 (38.5%)	2,142	858 (40.1%)	1.03 (0.93,1.13)	0.621	15–55 (41.5)
**Prevalence of ** ***P. falciparum***							
Unadjusted	2,106	302 (14.3%)	2,276	243 (10.7%)	0.76 (0.49,1.18)	0.221	11–55 (43.4)
Adjusted^d^	2,106	302 (14.3%)	2,276	243 (10.7%)	0.71 (0.46,1.11)	0.131	11–55 (43.4)
**24-MONTHS FOLLOW-UP**	2,468		2,619				
**Prevalence of anaemia** c							
Unadjusted	2,027	809 (39.9%)	2,173	910 (41.9%)	1.05 (0.91,1.21)	0.514	15–55 (41.6)
Adjusted	1,935	765 (39.5%)	2,027	842 (41.5%)	1.00 (0.90,1.11)	0.953	14–55 (39.5)
**Prevalence of ** ***P. falciparum***							
Unadjusted	2,001	169 (8.5%)	2,139	253 (11.8%)	1.42 (0.84,2.42)	0.192	15–55 (41.0)
Adjusted^d^	2,001	169 (8.5%)	2,139	253 (11.8%)	1.53 (0.89,2.62)	0.124	15–55 (41.0)

Results presented (i) for all children with outcome data (unadjusted) and (ii) for those with baseline measurements of each outcome and accounting for age, sex, and stratification effects (adjusted) as the primary pre-specified analysis. *N*, number of children eligible for follow-up (not withdrawn or deceased). Adjusted: for baseline age, sex, school mean exam score and literacy group (to account for stratification), and baseline measure of the outcome, where available; unadjusted: all children with outcome measures, not adjusted for any baseline or study design characteristics.

a Risk ratios (intervention/control) presented for binary outcomes (anaemia and *P. falciparum* prevalence) and are obtained from GEE analysis accounting for school-level clustering.

b Number and percentage with outcome.

c Age-sex specific anaemia was defined using age and sex corrected WHO thresholds of Hb: <110 g/l in children under 5 years; <115 g/l in children 5 to 11 years; <120 g/l in females 12 years and over and males 12 to 14.99 years old; and <130 g/l in males ≥15 years. All female adolescents are assumed to not be pregnant.

d Not including baseline *P. falciparum* infection.

### Effect of IST on Attention and Educational Achievement

At both 9- and 24-months follow-up, there was no statistical difference in mean scores for sustained attention between study groups in either class with adjusted mean difference (Adj.MD): −0.44, 95% CI −1.09 to 0.21, *p* = 0.180 and Adj.MD: 0.28, 95% CI −0.23 to 0.79, *p* = 0.283 for classes 1 and 5, respectively at the 24-month follow-up (Table 4). Similarly there was no significant difference between groups on scores for spelling in the older class at 9- and 24-month follow-ups (Adj.MD: −0.31, 95% CI −1.26 to 0.63, *p* = 0.515 and Adj.MD: 0.71, 95% CI −0.34 to 1.76, *p* = 0.183) nor for arithmetic at either follow-up (Table 5). However, at 9-months follow-up, children in the younger class in the intervention group had lower mean adjusted scores for the spelling task and the same trend was observed at 24 months (Adj.MD: −0.65, 95% CI −1.11 to −0.20, *p* = 0.005). Similarly at 24 months, in the younger class, children in the intervention group scored on average 0.60 points lower in the arithmetic assessments than children in the control group (Adj.MD: −0.60, 95% CI: −1.02 to −0.19, *p* = 0.005).

**Table 4:** Effect of the IST intervention at 9- and 24-months follow-up on sustained attention outcomes for younger (class 1) and older (class 5) children.

Outcome	Control (50 Schools)		Intervention (51 Schools)		Mean Difference^a^ (95% CI)	*p*-Value	Cluster-Size; Range (Mean)
	*N*	Mean (SD)^b^	*N*	Mean (SD)^b^			
**9-MONTHS FOLLOW-UP**							
**Class 1 (median age: 8, range: 5–15)**	1210		1,281				
**Sustained attention** c **(score: 0–20)**							
Unadjusted	1,070	8.48 (3.63)	1,162	8.43 (3.76)	−0.04 (−0.58 to 0.51)	0.895	8–27 (22.1)
Adjusted	1,030	8.52 (3.65)	1,144	8.43 (3.77)	−0.13 (−0.66 to 0.39)	0.623	5–27 (21.7)
**Class 5 (median age: 12, range: 8–18)**	1283		1,365				
**Sustained attention** d **(score: 0–20)**							
Unadjusted	1,180	13.38 (5.45)	1,231	13.35 (5.13)	−0.09 (−0.77 to 0.56)	0.799	8–30 (23.9)
Adjusted	1,178	13.38 (5.45)	1,221	13.40 (5.10)	−0.21 (−0.81 to 0.39)	0.490	8–30 (23.8)
**24-MONTHS FOLLOW-UP**							
**Class 1 (median age: 8, range: 5–15)**	1201		1,269				
**Sustained attention** c **(score: 0–20)**							
Unadjusted	960	13.45 (5.15)	1,059	13.20 (4.96)	−0.26 (−0.95 to 0.43)	0.456	8–26 (20.0)
Adjusted	923	13.49 (5.15)	1,041	13.18 (4.96)	−0.44 (−1.09 to 0.21)	0.180	4–25 (19.6)
**Class 5 (median age: 12, range: 9–18)**	1267		1,350				
**Sustained attention** d **(score: 0–20)**							
Unadjusted	1,007	14.22 (4.90)	1,052	14.66 (4.60)	0.40 (−0.14 to 0.94)	0.144	6–31 (20.4)
Adjusted	1,006	14.21 (4.90)	1,044	14.70 (4.58)	0.28 (−0.23 to 0.79)	0.283	6–29 (20.3)

Results presented (i) for all children with outcome data (unadjusted) and (ii) for those with baseline measurements of each outcome and accounting for age, sex, and stratification effects (adjusted) as the primary pre-specified analysis. *N*, number of children eligible for follow-up (not withdrawn or deceased). Adjusted: for baseline age, sex, school mean exam score and literacy group (to account for stratification), and baseline measure of the outcome, where available; unadjusted: all children with outcome measures, not adjusted for any baseline or study design characteristics.

a Mean difference (intervention-control) are obtained from GEE analysis accounting for school-level clustering.

b Mean score and SD at follow-up.

c Pencil tap test was conducted at baseline and single digit code transmission task was conducted at 9- and 24-months follow-ups.

d Double digit code transmission was conducted at baseline and both follow-ups.

**Table 5:** Effect of the IST intervention at 9- and 24-months follow-up on educational achievement (spelling and arithmetic) outcomes for younger (class 1) and older (class 5) children.

Outcome; *N* (%)	Control (50 Schools)		Intervention (51 Schools)		Mean Difference^a^ (95% CI)	*p*-Value	Cluster-Size; Range (Mean)
	*N*	Mean (SD)^b^	*N*	Mean (SD)^b^			
**9-MONTHS FOLLOW-UP**							
**Class 1 (median age: 8, range: 5–15)**	1,210		1,281				
**Spelling (score: 0–20)** c							
Unadjusted	1,068	11.70 (4.59)	1,162	10.47 (4.57)	−1.23 (−2.21 to −0.24)	0.015	8–27 (22.1)
Adjusted	1,060	11.69 (4.59)	1,133	10.49 (4.58)	−0.67 (−1.26 to −0.08)	0.026	8–27 (21.7)
**Arithmetic (score: 0–20)** d							
Unadjusted	1,071	4.21 (3.13)	1,162	4.04 (3.26)	−0.17 (−0.60 to 0.26)	0.433	8–27 (22.1)
Adjusted	1,069	4.21 (3.12)	1,143	4.07 (3.28)	−0.21 (−0.54 to 0.12)	0.214	8–27 (21.9)
**Class 5 (median age: 12, range: 8–18)**	1,283		1,365				
**Spelling (score: 0–75)** e							
Unadjusted	1,169	31.34 (12.61)	1,223	28.73 (12.36)	−2.73 (−5.26 to −0.19)	0.035	8–30 (23.7)
Adjusted	1,154	31.37 (12.60)	1,214	28.76 (12.34)	−0.31 (−1.26 to 0.63)	0.515	8–30 (23.4)
**Arithmetic (score: 0–30)** f							
Unadjusted	1,180	31.15 (5.49)	1,229	30.72 (5.17)	−0.49 (−1.40 to 0.42)	0.294	8–30 (23.9)
Adjusted	1,173	31.14 (5.50)	1,210	30.73 (5.17)	0.13 (−0.41 to 0.68)	0.629	8–30 (23.6)
**24-MONTHS FOLLOW-UP**							
**Class 1 (median age: 8, range: 5–15)**	1,201		1,269				
**Spelling (score: 0–20)** c							
Unadjusted	961	12.03 (3.05)	1,062	11.04 (3.49)	−0.97 (−1.54 to −0.40)	0.001	8–26 (20.0)
Adjusted	954	12.02 (3.05)	1,036	11.04 (3.50)	−0.65 (−1.11 to −0.20)	0.005	8–25 (19.7)
**Arithmetic (score: 0–30)** g							
Unadjusted	962	5.97 (3.05)	1,061	5.38 (2.97)	−0.59 (−1.08 to −0.10)	0.018	8–26 (20.0)
Adjusted	960	5.97 (3.04)	1,042	5.40 (2.97)	−0.60 (−1.02 to −0.19)	0.005	8–25 (19.9)
**Class 5 (median age: 12, range: 9–18)**	1,267		1,350				
**Spelling (score: 0–78)** e							
Unadjusted	1,010	35.28 (12.91)	1,060	33.97 (12.79)	−1.58 (−4.01 to 0.85)	0.202	6–31 (20.5)
Adjusted	996	35.33 (12.85)	1,052	34.04 (12.75)	0.71 (−0.34 to 1.76)	0.183	6–29 (20.3)
**Arithmetic (score: 0–30)** f							
Unadjusted	1,016	21.20 (5.47)	1,062	20.15 (5.68)	−1.07 (−2.15 to 0.00)	0.050	6–31 (20.6)
Adjusted	1,009	21.20 (5.48)	1,045	20.18 (5.69)	−0.49 (−1.32 to 0.34)	0.243	6–29 (20.3)

Results presented (i) for all children with outcome data (unadjusted) and (ii) for those with baseline measurements of each outcome and accounting for age, sex, and stratification effects (adjusted) as the primary pre-specified analysis. *N*, number of children eligible for follow-up (not withdrawn or deceased). Adjusted: for baseline age, sex, school mean exam score and literacy group (to account for stratification) and baseline measure of the outcome, where available; unadjusted: all children with outcome measures, not adjusted for any baseline or study design characteristics.

a Mean difference (intervention-control) for scores on spelling and arithmetic are obtained from GEE analysis accounting for school-level clustering.

b Mean score and SD at follow-up.

c The same class 1 spelling task was given at baseline, 9- and 24-months follow-ups, with different words used for the 24-month follow-up.

d Same addition task conducted at 9-months follow-up and at baseline, hence baseline adjustment is for the same task.

e The same class 5 spelling task was given at baseline, 9- and 24-months follow-ups, with different words used for the 24-month follow-up.

f Same arithmetic task conducted at baseline, 9- and 24-months follow-ups, with different sums used for the 24-month follow-up.

g Addition task conducted at baseline and arithmetic task containing addition, subtraction, multiplication, and division conducted at 24-months follow-up, hence baseline adjustment for different task.

### Surveillance for Adverse Effects

Active surveillance found that 4.5% (92/2,030) children reported one or more adverse effects within 2 days of receiving treatment, including headache (68; 3.3%), stomach ache (38; 1.9%), dizziness (17; 0.8%), vomiting (7; 0.3%), and pruritis (10; 0.5%). During the 24 months of follow-up, 11 children died: five in the intervention group and six in the control group. Cause of death was investigated and included yellow fever, heart defect, leukaemia, drowning, trauma, pneumonia, and paediatric HIV. In the intervention group, none of these deaths occurred within 30 days of the screening and treatment and therefore were not attributed to the intervention.

## Discussion

School-based malaria control is increasingly recognised as an important potential component for integrated school health packages [46]. However, as yet there is no consensus about the most effective malaria interventions for the alternative transmission settings. To our knowledge, we conducted the first cluster randomised trial of the impact of school-based IST of malaria. We failed to detect any overall benefit of IST using AL on the health, attention, or educational achievement of school children in this low-moderate malaria transmission setting.

The reasonably high follow-up rates of, on average, 87.0% and 79.4% at the first and second follow-ups, respectively, equal between groups at each follow-up, suggest sample bias was not responsible for the lack of impact observed. The higher proportion of children unavailable for baseline health assessments was driven by a few initially apprehensive schools [47], which were subsequently assessed throughout the study and included in the unadjusted analyses. The differential baseline prevalence of *P. falciparum* in those children available and unavailable for follow-up in the intervention group may reflect a higher proportion of withdrawal and absenteeism on screening and assessment days in schools in low transmission regions, where there was no treatment benefit. However, such a situation is unlikely to have masked any impact of IST as historical exposure and current parasite prevalence is highly predictive of subsequent malaria risk [48],[49], and as such these children were less likely to have been infected and thus gain any potential benefit from treatment over the study period.

The absence of apparent differences between study groups in relation to either *Plasmodium* infection or anaemia at 12 or 24 months is contradictory to predictions from simulation analyses of mass screening and treatment in a moderate transmission setting [26],[27]. One reason for these contrasting results may be the different coverage rates, where the simulations assumed 80% intervention coverage of the whole community in contrast to this study where the IST intervention covered two classes of the school populations only. In this low-moderate transmission setting less than 20% of children screened were eligible for treatment at each round. However, the lack of differential impact on anaemia observed when schools were stratified by baseline prevalence of *Plasmodium* infection (a proxy for transmission intensity) and by number of treatments received at the individual level, suggests there was no impact on long-term health even amongst the children receiving AL treatment.

A possible explanation for the lack of impact of IST on anaemia at the group or individual level is high, localised, rates of re-infection and acquisition of new infections between screening rounds allowing no time for haematological recovery, indicated by the remarkably similar percentage of children RDT positive at each screening round. The use of AL may have contributed to rapid re-infection rates as it affords short (14–28 days) post-treatment protection [50],[51]. Such a protection period would have provided extensive time at risk of acquiring new infections before the next round of IST at least three months later. A potential alternative would be dihydroartemisinin-piperaquine [52], which would afford a longer post-treatment prophylaxis period than AL between screening rounds and has recently been successfully evaluated as part of IPT in Uganda [53]. Additionally, increased frequency of screening, six times a year as opposed to three, could reduce the time at risk for parasite carriage and allow for haematological recovery, but would be logistically and financially prohibitive. The marked, but stable heterogeneity of *Plasmodium* infection observed over the two years (school-level prevalence range: 0%–75%) resulted in several schools experiencing no infection throughout all screening rounds, and a small sample of schools exhibiting repeatedly high proportions of RDT positive study children at each round. This heterogeneity, compounded by the large proportion of untested and therefore untreated asymptomatic carriers remaining in the communities, likely led to study children in localised hotspots being exposed to high risk of infection immediately after treatment [20]. Analyses of the stability of infection at both the school and the individual level, and the environmental correlates of such patterns, will be presented in a future paper.

The evaluation identified two further limitations of the IST approach. First, there was variability in RDT performance between screening rounds, with lowest RDT sensitivity during the dry season. However, diagnostic performance in this analysis was estimated assuming microscopy as a “gold standard,” and in light of concerns of the diagnostic accuracy of such reference tests, alternative methods of estimation for two or more malaria diagnostic tools in the absence of a “gold standard” have been suggested [54]–[56]. Additional analysis is underway to investigate diagnostic performance of RDTs and expert microscopy as well as the influence of individual, local transmission and seasonal factors during the two-year study period. The recent study conducted in Burkina Faso failed to show a significant reduction in parasitaemia in the dry season following community-wide screening and treatment campaigns in the previous dry season [29], suggesting that screening and treatment with RDTs is not sensitive enough to reduce transmission even when delivered in a mass campaign. The use of PCR would constitute a more sensitive tool, additionally detecting subpatent infections that contribute to transmission [57]–[59], but would be operationally challenging. Second, there was a decline in supervised treatment over time, as it became logistically difficult for children who were absent on screening day and subsequently treated on a repeat visit to be followed up on treatment day two and three by the nurse. Such children and/or their guardians and older siblings were given the full regimen with instructions on how to take the doses at home over the three days [60]. Altering the treatment supervision by the nurse from three days to the first day only would greatly reduce the cost of the IST intervention [61]. Although evidence indicates that unsupervised treatment is as effective at clearing parasitaemia as fully supervised treatment in clinical cases [62], unsupervised compliance may be lower when treating asymptomatic infection. Low efficacy of AL in the study is possible. No specific treatment efficacy evaluation was performed during this trial; however, although there is mixed evidence as to whether there is a slight decline in efficacy of AL in Kenya [63],[64], overall treatment success is thought to remain reasonably high.

In a region such as coastal Kenya, where food security is particularly low [65],[66] and malaria transmission is low-moderate, it is probable that factors such as long term nutritional status, short term access to food, and helminth infections are stronger contributors to the aetiology of anaemia in this setting [67] than parasitaemia. These factors would result in a limited impact on anaemia though a programme targeting malaria only, rather than a package containing a combination of school-feeding, deworming, and malaria control. This study thus contrasts with the previous IPT study conducted in Nyanza province, Kenya [24], where malaria is predicted to be the greatest contributor to anaemia [67], enabling a malaria control programme to have a large impact on anaemia directly.

Our finding of no significant differences between groups for sustained attention in either the younger or older classes at either follow-up is consistent with expectations, based on the lack of effect of IST on the assumed mediator, health. Likewise with the adjusted literacy and numeracy scores in the older class at both follow-ups, no significant differences between groups were found. However, in the younger class at both 9 and 24 months, there was an apparent negative effect of the IST intervention on literacy scores and on arithmetic scores at 24 months. This seemingly negative impact of IST was found only in the younger class, where the literacy intervention was implemented. As no statistical interaction between the two interventions was detected in the younger class, the differences between study groups cannot be attributed to an effect of the literacy intervention. Because of the multiple tests conducted, this finding could be due to chance. If we were to use a highly conservative Bonferroni correction for the 16 tests (two health and six education outcomes, all at two follow-ups) from adjusted models, the apparent negative effects on spelling and arithmetic would lie close to the updated significance level. Alternatively, these findings could demonstrate a negative effect of the by-term screening, involving an uncomfortable finger prick [68], with the intervention group experiencing increased apprehension of the finger prick during the education assessments as they associated the presence of our research team with the IST process [47], or reduced classroom attendance throughout the year in this group to avoid the IST intervention, or a combination. However, attendance measured at health and education assessment visits indicated no significant differences in attendance between the groups. Findings of negative educational or cognitive effects of health interventions are rare but not unprecedented [69] and suggest the need for experimental evaluations to test assumptions about the educational benefits of health programs. The finding of low overall achievement levels and minimal learning is consistent with the international literature and findings from Kenya [36]. The causes are well documented and include a lack of a culture of literacy, lack of effective teaching methods, poorly resourced teachers with large classes, poor health of children, and competition for children's time at home [70],[71].

Our study has a number of limitations. First, given the nature of the intervention, it was not possible to blind the parents, participants, or field officers delivering the IST intervention to experimental assignment, which could have led to a possible “John Henry” effect whereby children in the control group adjust their behaviour as they know they are not receiving the intervention, for example in risk aversion and treatment seeking behaviour. Biomedical and educational assessors were blinded where feasible. Second, study children's access to alternative malaria treatments outside of the school-based IST rounds was not monitored during the two years of the trial. However, due to the randomised design of the trial and the fact that the majority of infections in this age group and population were asymptomatic at assessment and screening points, we have no reason to suspect that study children's access to treatment outside of this trial differed greatly across study groups. Finally, the lack of multiple testing adjustments may have increased the possibility of type 1 error, and results should be interpreted in light of this possible error, but it is unlikely to have masked a beneficial effect of IST.

### Conclusion

In summary, our findings show there are no health or education benefits of implementing school-based IST with AL in a low to moderate transmission setting such as this study site, as a high proportion of children screened do not require treatment and those who do largely live in focal high transmission regions, where rapid re-infection occurs between screening rounds and results in no lasting gains from treatment. Nevertheless, our results do highlight a potential role for schools as screening platforms. School screenings using RDTs could provide an operationally efficient method to initially identify transmission hotspots for targeted community control [72]. School surveys have proved a useful platform for defining heterogeneities in *Plasmodium* transmission over large geographical areas in a more rapid and low cost manner than community surveys [73],[74]. The results from this study's screening rounds present a case for the use of schools in also depicting local transmission heterogeneities, which can be extrapolated to the local community [75] and aid in developing targeted community-wide comprehensive interventions, such as localised indoor residual screening and larviciding, with biennial school screenings used to monitor the success of these interventions. The use of schools in this way is a focus of our current research.

## Supporting Information

Alternative Language Abstract S1 **Spanish translation of the abstract by Jorge Cano Ortega.**
(DOC)

Alternative Language Abstract S2 **Swahili translation of the abstract by Carlos Mcharo and George Okello.**
(DOC)

Alternative Language Abstract S3 **French translation of the abstract by Birgit Nikolay and Fiona Majorin.**
(DOC)

Checklist S1 **CONSORT checklist.**
(DOC)

Protocol S1 **Study protocol.**
(PDF)

Table S1 **Baseline measures for 5,233 study children with missing 12-months follow-up health data versus those not missing 12-months follow-up health data across both the control and IST intervention groups.**
(DOC)

Table S2 **Baseline measures for 5,233 study children with missing 24-months follow-up health data versus those not missing 24-months follow-up health data across both the control and IST intervention groups.**
(DOC)

Table S3 **Results from missing data analysis for anaemia.** Effect of the IST intervention at 12- and 24-months follow-up on the primary health outcome of anaemia for study children combined using a longitudinal, random effects regression modeling approach. Results presented (i) for all children with either 12- or 24-months follow-up measurements of the outcome (unadjusted), (ii) for those with baseline measurements of the outcome and accounting for age, sex, and stratification effects as the primary pre-specified analysis, and (iii) for those additionally with baseline measures of parental education, SES, and baseline educational level (measured by baseline spelling) as further predictors of missingness.
(DOC)

Table S4 **Baseline measures for study children with missing 9-months follow-up education data versus those not missing 9-months follow-up education data across both the control and intervention groups.**
(DOC)

Table S5 **Baseline measures for study children with missing 24-months follow-up education data versus those not missing 24-months follow-up education data across both the control and intervention groups.**
(DOC)

Table S6 **Results from missing data analysis for sustained attention.** Effect of the IST intervention at 9- and 24-months follow-up on sustained attention outcomes for younger (class 1) and older (class 5) children combined using a longitudinal, random effects regression modeling approach. Results presented (i) for all children with either 9- or 24-months follow-up measurements of the outcome (unadjusted), (ii) for those with baseline measurements of the outcome and accounting for age, sex, and stratification effects as the primary pre-specified analysis, and (iii) for those additionally with baseline measures of parental education, SES, and baseline educational level (measured by baseline spelling) as further predictors of missingness.
(DOC)

Table S7 **Results from missing data analysis for spelling.** Effect of the IST intervention at 9- and 24-months follow-up on spelling outcomes for younger (class 1) and older (class 5) children combined using a longitudinal, random effects regression modeling approach. Results presented (i) for all children with either 9- or 24-months follow-up measurements of the outcome (unadjusted), (ii) for those with baseline measurements of the outcome and accounting for age, sex, and stratification effects as the primary pre-specified analysis, and (iii) for those additionally with baseline measures of parental education, SES, and baseline educational level (measured by baseline spelling) as further predictors of missingness.
(DOC)

Table S8 **Sensitivity analyses considering transfers across the study period.** Effect of the IST intervention at 12- and 24-months follow-up on health outcomes for study children. Results presented (i) for all children with either 12- or 24-months follow-up measurements of the outcome (unadjusted) with children who transferred schools excluded and (ii) for those with baseline measurements of each outcome and accounting for age, sex, and stratification effects as the primary pre-specified analysis with children who transferred schools excluded.
(DOC)

Table S9 **Analysis stratified by categories of** ***P. falciparum*** **prevalence at baseline.** Effect of the IST intervention at 12- and 24-months follow-up on the prevalence of anaemia, by baseline prevalence category of *P. falciparum* (control school prevalence estimated using 12-month follow-up data) with adjustment for age, sex, and stratification effects.
(DOC)

Table S10 **Analysis stratified by number of AL treatments received.** Effect of the IST intervention at 12- and 24-months follow-up within the IST intervention group by number of positive results and subsequent treatments received at the individual level.
(DOC)

Text S1 **Methods for the missing data models.**
(DOC)

## Abbreviations

Adj.MD: adjusted mean difference; Adj.RR, adjusted risk ratio; AL, artemether lumefantrine
GEE: generalized estimating equations
Hb: haemoglobin
ICC: intraclass correlation coefficient
IPT: intermittent preventive treatment
IQR: inter-quartile range
IST: intermittent screening and treatment
RDT: rapid diagnostic test
SD: standard deviation
SES: socioeconomic status
