# Supplementary material for: Impact of Intermittent Screening and Treatment for Malaria among School Children in Kenya: A Cluster Randomised Trial
Source: PLoS Med. 2014 Jan 28;11(1):e1001594. doi: 10.1371/journal.pmed.1001594 (PMC3904819; doi:10.1371/journal.pmed.1001594)
Supplement: Alternative Language Abstract S1 — Spanish translation of the abstract by Jorge Cano Ortega. (DOC) [file pmed.1001594.s001.doc]

**Impacto del cribado intermitente y el tratamiento de la malaria entre los niños en edad escolar en Kenya: ensayo aleatorizado por conglomerados**

**Antecedentes**. Mejorar la salud de los niños en edad escolar puede aportar importantes beneficios para el desarrollo cognitivo y el rendimiento educativo. Sin embargo, existen pocas evidencias experimentales de los beneficios de intervenciones alternativas contra la malaria en las escuelas o cómo el impacto de dichas intervenciones varían según la intensidad de la transmisión de la malaria. Nosotros investigamos el efecto del diagnóstico y el tratamiento intermitente (IST) de la malaria en la salud y la educación de los escolares en un área de baja a moderada transmisión de la malaria.

**Métodos y Resultados**. Se llevó a cabo un ensayo aleatorizado por conglomerados con 5.233 niños de 101 escuelas primarias públicas en la costa sur de Kenia durante el periodo 2010-2012. La intervención se administró a niños seleccionados al azar de las clases 1º y 5º que fueron seguidos durante 24 meses. Durante el curso escolar, los niños fueron examinados por trabajadores de salud utilizando test de diagnóstico rápido de la malaria (RDTs) y los niños, con o sin síntomas de la malaria, que resultaron RDT-positivos fueron tratados con un régimen de seis dosis de arteméter+lumefantrina (AL). Dada la naturaleza de la intervención, el ensayo no fue ciego. Los principales indicadores del estudio fueron la anemia y la atención continuada en el aula. Como indicadores secundarios se midió la parasitemia de los niños con malaria y el rendimiento escolar. Los datos fueron analizados en base a la intención de tratamiento. El estudio se ha registrado en ClinicalTrials.gov, NCT00878007.

Durante el período de intervención, un promedio del 88,3% de los niños en las escuelas incluidas en el estudio fue evaluado en cada ronda, de los cuales el 17,5% fue RDT- positivo. El 80,3% de los niños en el grupo control y el 80,2% en el grupo de intervención fueron seguidos hasta el 24º mes. No se observó ningún efecto del ITS sobre la prevalencia de anemia ó la infección por *P. falciparum* tanto a los 12 como a los 24 meses; Razón de riesgo ajustada (Adj.RR): 1,03 (IC95%: 0,93-1,13), p = 0,621 y Adj.RR: 1.00 (IC95%: 0,90-1,11) p = 0,953, respectivamente, o en los resultados sobre la atención en el aula. No se observó ningún efecto del IST en el logro educativo en los mayores (clase 5º), pero si un aparente efecto negativo en los resultados de las pruebas de ortografía en los más jóvenes a los 9 y 24 meses, y en los resultados de las pruebas aritméticas a los 24 meses.

**Conclusión**. El tratamiento intermitente, tal y como ha sido aplicado en este estudio, no ha mostrado ser eficaz en la mejora de la salud o la educación de los escolares en la costa sur de Kenia. Las posibles razones serían la marcada heterogeneidad geográfica en la transmisión de la malaria, la rápida tasa de reinfección después del tratamiento con AL, la variable fiabilidad de los RDTs y la contribución relativa de la malaria a la etiología de la anemia en este contexto.
