# Supplementary material for: Impact of Intermittent Screening and Treatment for Malaria among School Children in Kenya: A Cluster Randomised Trial
Source: PLoS Med. 2014 Jan 28;11(1):e1001594. doi: 10.1371/journal.pmed.1001594 (PMC3904819; doi:10.1371/journal.pmed.1001594)
Supplement: Alternative Language Abstract S2 — Swahili translation of the abstract by Carlos Mcharo and George Okello. (DOC) [file pmed.1001594.s002.doc]

**Athari za Upimaji na matibabu ya malaria kati ya wanafunzi wa shule za msingi: Utafiti wa bahati na sibu kwa makundi.**

**Muhtasari**

**Utangulizi.** Kuboresha afya ya watoto wenye umri wa kuenda shule kunaweza kuleta ubora katika kusoma na kuelewa. Hata hivyo, kuna ushahidi mchache kuhusu manufaa ya njia mbadala za kukinga malaria au vile athari za kukinga malaria zinahusiana, kulingana na wingi au uchache wa maambukizi ya malaria. Tulitafiti athari za kupima na kutibu malaria wa mara kwa mara kwa afya na masomo kwa wanafunzi kwa eneo lililoko na mkurupuko kadri wa ugongwa wa malaria.

**Mbinu na Matokeo.** Utafiti wa makundi uliopeana nafasi sawa kuchagua wale wanaoshiriki ulifanyika na ulihusisha wanafunzi 5,233 kutoka shule 101 za uma pwani kusini mwa Kenya kuanzia mwaka wa 2010 hadi mwaka wa 2012. Utafiti huu ulifanywa kwa wanafunzi wa darasa la 1 na la 5 waliochaguliwa kwa njia iliyowapa nafasi sawa na wakafuatiliwa kwa miezi 24. Kila muhula, wanafunzi walipimwa na wauguzi wa kitengo cha afya kwa kutumia kifaa cha kupima malaria papo hapo (kwa Kimombo RDT) na wanafunzi waliopatikana kuwa na ugonjwa wa malaria (walio na wasio na dalili za malaria) walipewa dozi sita za dawa ya malaria inayojulikana kama AL (Artemeter Lumefantrine). Kufuatia hali ya utafiti, uchunguzi huu haukuficha makundi yaliyowekwa washiriki. Matokeo msingi ya utafiti huu yalikuwa ni upungufu wa damu mwilini na kumakinika kabisa darasani. Matokeo mengineyo yalikuwa ni viini vya malaria na mafanikio kielimu. Uchambuzi wa matokeo ya utafiti huu ulijumlisha wote waliojiunga na utafiti huu hata wale waliotamatisha kuhusika kwao kabla ya utafiti kukamilika. Utafiti ulisajiliwa na ClinicalTrials.gov, NCT00878007.

Wakati wa utafiti huu, kwa wastan, asilimia 83.3 ya wanafunzi walioshiriki kwa mradi walipimwa mara kwa mara. Kaati ya hawa, asilimia 17.5 walipatikana na viini vya malaria. Asilimia 80.3 ya wanafunzi ambao hawakushiriki katika kupimwa na kutibiwa katika mradi, walifuatiliwa kwa miezi 24. Hakuna athari zozote zilionekana katika kupima na kutibu malaria kwa uambukizo wa malaria wala viini vya malaria, ulioonekana katika miezi wa 12 au 24 (Kukidhi kutowiana (yaani Adjusted risk ratio) (Adj.RR): 1.03, 95% Kiwango cha kuthibitisha (yaani Confidence Intervals) (CIs): 0.93, 1.13 p=0.621 na Adj.RR: 1.00, 95%CIs: 0.90, 1.11 p=0.953) kufuatilia, au matokeo ya umakinifu darasani. Hakuna athari za kupima na kutibu watoto mara kwa mara zilizoonekana kwa matokeo ya masomo katika darasa la tano, lakini athari ya kupungua ilionekana katika matokeo ya imla ya wanafunzi wa darasa la 1 kwa miezi wa 9 na wa 24 na kwa matokeo ya hesabati katika miezi wa 24.

**Hitimisho.** Katika muktadha huu nchini Kenya, kupima na kutibu mara kwa mara kama vile ilivyofanywa katika huu mradi si ya kuboresha afya wala masomo ya wanafunzi. Sababu za kukosekana kwa kuboreka kwa afya na masomo ni ukawaida wa maambukizi, na uharaka wa maambukizi ya mara ya pili baada ya matibabu kutumia dawa ya AL, msukosuko wa uaminifu kwa vifaa vya upimaji vya malaria papo hapo (yaani RDTs), na changio la malaria kwa ukosefu wa damu mwilini kwa eneo hili.

**Maneno ya muhimu:** malaria, *Plasmodium falciparum*, Kupima na kutibu malaria, shule, artemether-lumefantrine, Africa.
