# Supplementary material for: Impact of Intermittent Screening and Treatment for Malaria among School Children in Kenya: A Cluster Randomised Trial
Source: PLoS Med. 2014 Jan 28;11(1):e1001594. doi: 10.1371/journal.pmed.1001594 (PMC3904819; doi:10.1371/journal.pmed.1001594)
Supplement: Alternative Language Abstract S3 — French translation of the abstract by Birgit Nikolay and Fiona Majorin. (DOC) [file pmed.1001594.s003.doc]

**Impact du dépistage et du traitement intermittent du paludisme chez les enfants d’âge scolaire au Kenya: un essai randomisé par grappes**

**Résumé**

**Contexte.** Améliorer la santé des enfants d'âge scolaire peut apporter des avantages substantiels pour le développement cognitif et la réussite scolaire. Cependant, il existe peu de preuves expérimentales des avantages des interventions alternatives contre le paludisme en milieu scolaire ou de la façon dont les impacts des interventions varient selon l'intensité de la transmission du paludisme. Nous avons étudié l'effet du dépistage et du traitement intermittent (IST) du paludisme sur la santé et l'éducation des enfants d'âge scolaire dans une zone de transmission faible à modérée du paludisme.

**Méthodes et résultats.** Un essai randomisé par grappes a été réalisé avec 5233 enfants dans 101 écoles primaires publiques sur la côte sud du Kenya, en 2010-2012. L'intervention a été délivrée à des enfants choisis de manière aléatoire dans les classes de première et cinquième qui ont été suivis pendant 24 mois. Une fois par semestre scolaire, les enfants ont été examinés par des travailleurs de la santé publique en utilisant des tests diagnostiques rapides du paludisme (TDR), et les enfants (avec ou sans symptômes du paludisme) diagnostiqués comme positif par le TDR ont été traités avec six doses d'artéméther - luméfantrine (AL). Compte tenu de la nature de l'intervention, l’essai n'a pas été aveugle. Les résultats primaires étaient l’anémie et l’attention soutenue. Les résultats secondaires étaient la parasitémie du paludisme et la réussite scolaire. Les données ont été analysées en intention de traiter. L'étude est enregistrée sur ClinicalTrials.gov, NCT00878007.

Au cours de la période d'intervention, une moyenne de 88.3 % des enfants dans les écoles d'intervention ont été dépisté à chaque semestre, dont 17.5 % étaient positifs selon les TDR. 80.3 % des enfants dans le group contrôle et 80.2 % dans le groupe d'intervention ont été suivis au bout de 24 mois. Aucun impact de l’IST du paludisme a été observé sur la prévalence de l'anémie ou du paludisme à P. falciparum à 12 ou 24 mois (risque relatif ajusté (Aj.RR): 1.03, intervalle de confiance à 95 % (IC) : 0.93, 1.13, p = 0,621 et Aj.RR : 1.00, IC à 95% : 0.90, 1.11, p= 0.953) respectivement, ou sur les résultats d'attention soutenue en classe. Aucun effet de l’IST n’a été observé sur la réussite scolaire dans la classe des plus âgés, mais un effet négatif apparent a été mesuré sur les résultats d'orthographe dans la classe des plus jeunes à 9 et 24 mois et sur ​​les résultats arithmétiques à 24 mois.

**Conclusion.** Dans ce cadre au Kenya, l’IST délivré comme dans cette étude n'est pas efficace pour améliorer la santé ou l'éducation des enfants d'âge scolaire. Les raisons possibles de l'absence d'impact sont l'hétérogénéité géographique marquée de la transmission, la rapidité de réinfection après le traitement avec AL, la fiabilité variable des TDR et la contribution relative du paludisme à l'étiologie de l'anémie dans ce cadre.

**Mots-clés:** paludisme, Plasmodium falciparum, dépistage et traitement intermittent, écoles, artéméther - luméfantrine, Afrique
