# Supplementary material for: Impact of Intermittent Screening and Treatment for Malaria among School Children in Kenya: A Cluster Randomised Trial
Source: PLoS Med. 2014 Jan 28;11(1):e1001594. doi: 10.1371/journal.pmed.1001594 (PMC3904819; doi:10.1371/journal.pmed.1001594)
Supplement: Protocol S1 — Study protocol. (PDF) [file pmed.1001594.s005.pdf]

1. Title of project

Impact of malaria prevention on health and education in Kenyan schoolchildren

2. Investigators and Institutional Affiliations

Principal investigator:
Dr. Simon Brooker[1]

Co-investigators:
Dr. Joseph Kiambo Njagi[2]*
Prof. Benson Estambale[3]*
Dr. Charles Mwandawiro[4]
Dr. Siân Clarke[5]*
Dr Dr. Matthew Jukes[6]*
Dr. Margaret Dubeck[6]*
Mr George Okello[1]
Dr, Caroline Jones[7]

[1] KEMRI/Wellcome Trust Collaborative Programme, Nairobi, Kenya
[2] Division of Malaria Control, Ministry of Public Health and Sanitation, Nairobi, Kenya
[3] Institute of Tropical and Infectious Diseases, University of Nairobi, Kenya
[4] ESACIPAC, Kenya Medical Research Institute, Nairobi, Kenya
[5] London School of Hygiene and Tropical Medicine, UK
[6] Harvard University, USA
[7] KEMRI/Wellcome Trust Collaborative Programme, Kilifi, Kenya

*CVs of non-KEMRI staff are attached (Appendix 1)

3. Abstract

The Government of Kenya is committed to improving the education of its children, and recognizes the importance of child health for educational achievement. While malaria represents one of the main health problems afflicting Kenyan schoolchildren, the evidence base for policy development and programme implementation for school-based malaria control remains inadequate. A recent study in western Kenya showed that delivering intermittent preventive treatment (IPT) to schoolchildren improved rates of anaemia and classroom concentration, but did not improve school performance. This study aims to (i) investigate the impact of a related malaria prevention strategy, intermittent screening and treatment, on health and education among schoolchildren in a different malaria transmission setting and (ii) determine the interaction between health and improved literacy instruction. The study will be undertaken in 100 randomly selected primary schools in Kwale District. IST will consist of all children being screened using rapid diagnostic tests (RDTs) for malaria once a term (thrice yearly). Children (with or without malaria symptoms) found to be RDT-positive will be treated with artemisinin-based combination therapy (ACT). Screening and treatment will be administered by district health workers. The education intervention will involve a programme of training for primary school teachers to improve literacy instruction. The study is designed to detect a 25% reduction in anaemia and an improvement of 0.2 standard deviations in mathematics and literacy tests. Additional outcomes will also be measured including malaria parasitaemia, classroom attention and school attendance. Health and educational outcomes will be assessed before intervention and twelve and twenty four months later. Information will also be collected on in-vivo parasitological efficacy of treatments and the cost-effectiveness of intervention. The findings of this study will provide critical information on the effectiveness of interventions to reduce the health and education burden of malaria among school-aged children.

4. Background

Malaria poses an enormous public health burden among the population of Kenya, and several donors are supporting the up-scaling of currently available malaria interventions. Typically, these efforts focus on children under five years and pregnant women because they bear the brunt of morbidity and mortality. However, older children, including those attending school, are also at risk of mortality and morbidity (Lalloo 2006). In addition, malaria greatly contributes to school absenteeism, poor cognition, learning and school performance (Brooker et al., 2000; Holding & Snow, 2001; Lalloo, 2006).

Despite this burden among schoolchildren, there is currently no consensus as to the appropriate approach to malaria control in schools, with relevant intervention strategies likely to vary according to patterns of malaria transmission (Brooker et al., 2008). A promising school-based intervention strategy, already proven effective for improving the health of women in pregnancy, is intermittent preventive treatment (IPT). In a recent study in western Kenya, the mass administration of a full therapeutic course of an anti-malarial drug to schoolchildren once a term, irrespective of infection status, dramatically reduced malaria parasitaemia, almost halved the rates of anaemia, and significantly improved cognitive ability (Clarke et al. 2008; Temperley et al., 2008). The withdrawal in Kenya in 2009 of sulfadoxine-pyrimethamine (SP) and amodiaquine (AQ) from use means that these drugs can no longer been used for IPT. An alternative approach is intermittent screening and treatment (IST), a malaria prevention strategy recently identified in the Kenyan National Malaria Strategy, 2009-2017 as a possible school-based strategy in the newly launched *Malaria Free School Initiative*.

The proposed study will investigate the health and educational impact of school-based IST in a different epidemiological setting in Kenya, where other important contributors to anaemia in school-aged children, including poor nutrition and helminth infections, are important. In addition, the study will investigate a number of scientific and operational issues which yield generate valuable evidence to assist in the formulation of policy recommendations for Kenya and for sub-Saharan Africa.

a) Malaria in schoolchildren

Although all-cause mortality rates are lowest among school-age children, it is estimated that malaria causes up to 50% of all deaths in this age group in Africa (Snow et al., 2003). Morbidity studies undertaken in western Kenya suggest that between 20% to 50% of schoolchildren living in areas of stable transmission experience clinical malaria attacks each year (Clarke et al., 2004). During malaria epidemics, the incidence of malaria in schoolchildren in areas of unstable transmission may be six times higher than in areas of stable transmission (Clarke et al., 2004). Malaria (both clinical and asymptomatic malaria) is also a major cause of anaemia among school-age children (Kurtzhals et al., 1999), and efforts to control malaria among this age group can dramatically improve haemoglobin levels (Geerligs, Brabin, & Eggelte, 2003).

b) Educational impact of malaria

In Kenya, primary schoolchildren miss 11% of school days per year because of malaria, and secondary schoolchildren 4% of school days (Leighton & Foster, 1993), equivalent to 4 to 10 million schooldays lost annually (Brooker et al. 2000). Evidence from Sri Lanka found that school performance is related to the cumulative impact of previous malaria attacks (Fernando et al., 2003a) and that weekly chemoprophylaxis with chloroquine improved school examination scores (Fernando et al., 2003b). This impact of malaria was mediated, at least in part, by children's absence from school due to clinical malaria, and it remains unclear from this study whether malaria prevention improved learning processes in the classroom.

In recent study in western Kenya (Clarke et al. 2008) we examined the cognitive processes involved in learning and found that children's sustained attention was improved by IPT. However, this improvement was not translated into improved education achievement over the 12 month course of the study. Possible explanations for this finding are short follow-up (one year) or that children were not given the educational resources (such as textbooks) or a sufficient period of effective instruction to learn effectively during the time course of the study. It is highly plausible that the improved sustained attention we observed in our first study would translate into improved educational achievement, particularly in the early grades of school. Recent evidence (Blair & Razza, 2007) suggests that executive function skills, such as regulation and attention, are particularly important for early achievement. To achieve a measurable impact on education, it may also be necessary to improve teaching methods in order to capitalise on any improvements in health status of schoolchildren following malaria control

In the proposed study we will follow children up for a longer period of time (at least two years) and will also conduct an intervention to improve the quality and quantity of literacy instruction. This will help ensure that children have the opportunity to apply improved cognitive skills and to learn effectively. It will also give us the opportunity to study the interaction between literacy instruction and malaria prevention to investigate whether enhanced instruction is more effective when children are more healthy, and to study the cognitive processes by which this interaction takes place. The role of reading abilities and the teaching methods to develop them is recognized for promoting student success and continual enrollment in school (Jimerson et al., 2000; NICHD, 2000).

c) Literacy development

As in many countries, Kenyan students begin to learn to read in their first year of schooling. Yet, about half of Kenya students who enter primary school drop out before they reach grade 8 (Muthwii, 2004). The drop-out rate could be partially due to low performance in literacy skills (Nzomo et al., 2001).

Kenyan government policy does not mandate that a single teaching method should be used to teach reading (MOEST, 2001). Instead, the policy suggests that the teaching methods should meet the students' learning needs and the objective for the lesson (Commeyras & Inyega, 2007). However, the current approach does not help all enrolled students to learn to read. Nzomo et al. (2001) concluded that about 35 percent of Kenyan students did not reach the designated minimum mastery level of reading and about 77 percent did not reach the desirable level of reading. An intervention to improve the quality of classroom instruction will be designed to ensure that students benefit from literacy instruction that is understood to promote reading acquisition.

Evidence suggests that the critical components of successful literacy acquisition can be taught so that students learn to read more efficiently (Adams, 1990; August & Shanahan, 2006; Snow et al., 1998). When teachers develop their students' oral language skills (e.g., phonological awareness & vocabulary) and teach the relationship between letters and sounds in a systematic and explicit fashion, their students have the foundation for successful word recognition and reading comprehension. Kenyan teachers will be provided with in-service to support their delivery of these foundational literacy skills as well as their assessment these skills in order to optimize student learning.

5. Justification for Study

The Government of Kenya is committed to improving the education of its children, and recognizes the importance of child health for educational achievement. While malaria represents one of the main health problems afflicting Kenyan schoolchildren, the evidence base for policy development and programme implementation for school-based malaria control remains inadequate. A recent proof-of-principle trial in western Kenya has demonstrated the potential of delivering intermittent preventive treatment (IPT) to schoolchildren in order to improve their health and education. In light

of the promising results from this study, it is important to further investigate the benefits of malaria prevention in schools. A change in drug policy in Kenya means that IPT with SP and AQ is no longer a viable strategy, but that there is an important need to evaluate IST, as outlined in the revised National Malarai Strategy, 2009-2017. There is also the need to better understand the educational benefits of malaria prevention before clear policy recommendations can be made. This involves assessing the impact of the alternative strategy of IST on outcomes, such as children's ability to read, which have clear relevance to the aims of education systems. It also involves assessing the extent to which IST increases the effectiveness of improved classroom instruction. The current study will explore whether it is necessary to improve teaching methods in order to capitalise on any improvements in health status of schoolchildren following malaria control. This understanding will yield important information for both heath and educational planners as they plan malaria control in school.

6. Null hypothesis

School-based malaria control using intermittent screening and treatment will not reduce rates of anaemia or improve educational outcomes in Kenyan schoolchildren, when compared to a placebo.

In addition, a programme of training for primary school teachers to improve literacy instruction will not improve literacy rates and there will be no interaction between the malaria intervention and the education intervention, such that learning will not be improved when teaching is effective and children are healthy.

7. Objectives

a) Overall objective:

To evaluate the impact of school-based malaria intervention using intermittent screening and treatment (IST) to reduce rates of anaemia among schoolchildren and hence improve classroom attention, school attendance and educational achievement of children in school.

b) Specific objectives:

1. To evaluate the efficacy of intermittent screening and treatment (IST) in improving haemoglobin concentration of schoolchildren.

2. To evaluate the efficacy of IST in reducing rates of asymptomatic malaria parasitiaemia of schoolchildren.

3. To evaluate the efficacy of IST in improving classroom attention, school attendance, and educational achievement of children in school.

4. To evaluate the impact of a programme of training for primary school teachers to enhance literacy instruction in improving literacy rates of schoolchildren.

5. To determine whether malaria and education interventions work synergistically together, such that learning is improved only effective teaching is effective and children are healthy to benefit from it.

6. To analyse the cost-effectiveness of IST in improving anaemia and education.

8. Design and Methodology

i) General Study design

This study will be a factorial-design, cluster-randomised, to assess the impact of IST and enhanced literacy instruction by teachers on the health and educational achievement of healthy schoolchildren. This study is one of two studies investigating the health and educational impact of malaria control, with a parallel study planned in Senegal, where malaria transmission is intense but highly seasonal.

The target population in this study includes children attending primary schools in Kenya. The accessible population includes the children attending the participating primary schools in standards 1-7 in Kwale district. The unit of analysis is the school. Schools will be randomized to one of four groups, receiving either the IST intervention alone, the education intervention alone, the IST and education interventions combined, or neither intervention. The first two arms would evaluate the impact of IST. The final two arms would investigate the hypothesis that IST helps children benefit from improved quality of education. Health and educational outcomes will be assessed before intervention and twelve and twenty four months later. The impact of each intervention will be estimated for education outcomes and health outcomes in separate multiple regression models. An interaction term will be included to identify any synergistic effects between the two interventions.

The survey data will be collected over two years – see section 10. Development and testing of survey instruments will take place in 2008. Baseline data collection and intervention activities will commence in January 2009, with follow-up surveys planned in 2010 and 2011. The study will be conducted by the Kenya Medical Research Institute in collaboration with the Division of Malaria Control in the Ministry of Public Health and Sanitation, University of Nairobi, the London School of Hygiene and Tropical Medicine, and Harvard University.

ii) Outcome measures

Primary outcomes:

1. Prevalence of anaemia
2. Education achievement assessed by a battery of tests of reading, writing and arithmetic

Secondary outcomes:

3. Prevalence of malaria parasitemia
4. Concentration as assessed by classroom-based tests of sustained attention
5. Classroom behavior as assessed by teacher ratings of children's inattentive and hyperactive-impulsive behaviors.
6. School attendance as assessed by class attendance registers
7. Examination results as assessed by government examination scores

iii) Study sites

The study will take place in rural primary schools in Kwale district, a hot and relatively dry area on the Kenyan coast. Continuous precipitation supports intense year-round transmission, with two seasonal peaks in malaria cases reflecting the bimodal rainfall pattern, with the heaviest rainfall typically occurring between April and June, with a smaller peak in October and November each year. Most malaria is caused by *Plasmodium falciparum*. A recent survey among 25 schools in 2008 found that the prevalence of malaria infection among schoolchildren in Kwale district was 9% (ranging from 1-28% by school), and 20% (3-37% by school) of children are anaemic (Brooker and Mwandawiro, unpublished data). Helminth infections are also common with 38% of schoolchildren estimated to be infected with hookworm and 41% infected with *Schistosoma haematobium*.

Kwale district also has some of the poorest educational indicators in the country, with the district having the worst KCPE examination scores for the last three years running.

iv) Study populations

Children enrolled in participating schools will be assessed for the following eligibility criteria:

1. **Inclusion criteria**
    a. Pupil enrolled at participating schools in standards 1-5
    b. Provision of informed consent from parent or guardian
    c. Provision of assent by student

2. **Exclusion criteria**
    a. Pupils unwilling to participate in the study
    b. Known allergy or history of adverse reaction to study medications
    c. Known or suspected sickle-cell trait. These children will be referred to testing and/or clinical management as per national guidelines

v) **Sample size determination**

Power analysis was adjusted for clustering (Hayes & Bennett, 1999) and focused on two main outcomes of interest: the percentage of children who are anaemic (haemoglobin <110 g/L); and education achievement assessed by a battery of tests of reading, writing and arithmetic.

For anaemia, assuming a baseline prevalence of 20% (as found in the 2008 school surveys in Kwale), an intraclass correlation of between 0.025 and 50 children sampled per school, a sample size of 21 schools with a total of 1050 children of all ages in each arm was estimated to provide 80% power to detect a 25% reduction in anemia in the intervention group compared to placebo at 5% level of significance. This equates to 84 schools and 4200 children.

For educational achievement tests, an overall sample size of 100 schools with 50 children per school (5000 children overall) is sufficient to detect an effect size of 0.2 standard deviation (SD) assuming an intra-class correlation of 0.2 (ICC varied from 0.1 to 0.2 with mathematics and literacy tests in Class 2 in 210 schools in Western Kenya; ICC is expected to be lower in Class 1) and a correlation between baseline and final test scores of 0.7. This sample size is sufficient to detect an effect size of 0.15 SD for concentration tests, which have a lower intra-class correlation (0.07 in the previous study in Bondo).

vi) Randomization and treatment assignment and allocation

Schools will be randomly assigned to one of the four intervention groups using a pre-defined stratified randomization procedure. A computer generated randomization list will be created by a member of the project who will not be directly involved in the conduct of the study. Sealed copies of the original randomization lists and documentation of the procedure used to generate the lists will be stored in KEMRI offices in Nairobi. Prior to the onset of the study, sealed copies of the randomization lists will be distributed to the member of the study team responsible for treatment allocation.

Schools will be stratified into five groups according to school examination performance in previous years, to account for differences in school quality and socio-economic environment. Five schools will be randomly selected from each school-performance stratum, and within each stratum schools were randomly allocated to 1 of 5 coded drug groups using block randomization according to the randomization listing.

vii) Study interventions

In this study, two interventions will be provided: i) intermittent screening and treatment (IST) for malaria and ii) a programme of training for primary school teachers to improve literacy instruction.

The malaria prevention strategy will involve all children being screened using rapid diagnostic tests (RDTs) for malaria once a term (thrice yearly). Children (with or without malaria symptoms) found

to be RDT-positive will be treated with artemisinin-based combination therapy (ACT). Screening and treatment will be administered by district health workers once a school term, observed by the evaluation research team.

An education intervention will be conducted in half of the schools receiving IST and half of the control schools. The intervention is designed to improved early grade literacy instruction. Recent evidence (Snow et al., 1998) suggests that relatively simple steps can be taken by teachers in order to improve the development of children's literacy in the early grades. It is particularly important to develop students' oral language skills (e.g., phonological awareness & vocabulary) and to teach the relationship between letters and sounds in a systematic and explicit fashion. Such instructional techniques are encouraged in the current Kenyan curriculum but teachers face many challenges in implementing them systematically and effectively. The aim of the instructional intervention will be to support teachers in implementing these teaching methods. Specific interventions will be design based on further consultation with all stakeholders and ongoing class observations in Kwale but may include training on (i) how to monitor students' progress in large classes (ii) developing and using instructional materials for reading (iii) lesson planning for explicit teaching of letter-sound relationships (iv) instructional techniques for large classes.

viii) Survey investigations

Survey tools and procedures have been used previously to estimate the health and educational impact of IPT in Kenyan schoolchildren (Clarke et al., 2008). These standard tools and operating procedures have been adapted based on previous collective experience. These are detailed below and summarized in Table 2.

Table 2. Outcome measurements

Outcome	Baseline	Follow-up 1	Follow-up 2
Consent			
Hb concentration			
Malaria parasitaemia			
Child literacy and numeracy			
School attendance			
Behavioural assessments			
Socio-economic and educational covariates			
Soil-transmitted helminths			
Urinary schistosomiasis			

Seeking consent: Selected schools will be visited one month prior to the survey date to have the purpose of the survey explained to the head teacher and school committee, and informed parental consent will be sought from the parents/guardians of children. See section 11. Parents of included children will be asked a series of questions including known parental education, known reactions of their children to anti-malarials, and location of household (Appendix 2). This information will used to (i) help trace children absent from school during follow-up and (ii) control for potentially confounding variables in the data analysis.

Survey procedures: Seven staff travelling in a single vehicle will visit each school: one supervisor; three laboratory technicians; one laboratory assistant; one nurse; plus one driver. An initial meeting will be held with the head teacher. In order to minimize disruption, one class will be selected at a time. Two education testers will assess cognitive function and educational achievement in the week before the arrival of the health team.

A series of questions will be asked of each randomly selected participating child including: age, fever on the day of the survey, basic household assets indicators, source of potable water in the homestead, use of mosquito nets treated with insecticide (Appendix 3). This information will used to control for potentially confounding variables in the data analysis.

On the day of enrollment to the study, children will have their height measured to the nearest 0.1 cm using a portable fixed base stadiometer and weight measured to the nearest 0.1 kg using an electronic balance.

No assessment of malaria parasitaemia and anaemia will be made **at baseline**. However, to measure success of the randomization, children from classes 6 and 7, not enrolled in the study, will be asked to provide a finger-prick blood sample for the preparation of a thick and thin blood smear for malaria investigation. The same finger prick sample will be used at the point of survey to record haemoglobin concentrations using a portable spectrophotometer.

All enrolled children in classes 1 to 5 will be asked to provide a finger-prick blood sample at **follow-up** to assess malaria parasitaemia and anaemia.

For those children with a reported fever or a history of fever in the last 48 hours, a drop of blood will be used to perform a rapid malaria test (OptiMal). Any child with a positive test and symptoms will be treated using artemether-lumefantrine (Co-artem) on the day of the survey according to national guidelines or referred to the nearest health facility if necessary. Transportation costs will be provided and an agreement will be reached with facilities to waive appropriate drug costs. Children identified as severely anaemic (haemoglobin levels < 8 g/dL) will receive ferrous sulphate according to national guidelines.

As part of a national school-based deworming, all schools in Kwale district would have received mass treatment for STH infection using albendazole [400mg]. Therefore, no anthelmintic treatment will be provided in the present study. During the final follow-up pots for stool samples will be distributed to all selected children who will be asked to return the following day with a sample. Children will also be asked to provide a urine sample. These results will be used to assess the need for further anthelmintic treatment, in accordance with national guidelines: mass treatment for STH infection using albendazole [400mg] in schools where prevaldnce exceeds 50%; mass treatment for *S. haematobium* using praziquantel [40 mg/kg] in schools where prevalence exceeds 50%; in remaining schools, only those found to be infected with *S. haematobium* will be treated.

ix) Education and cost-effectiveness assessments

Pre- and post-intervention education assessments will be performed in each school. This will involve a number of visits per year to each school. The first visit will occur one week prior to the biomedical assessments and will commence with classroom tests of attention for 30-60 minutes in standard 1 and individual assessments of pre-literacy and pre-numeracy skills. A team of 4 people can assess 60 children (2 classes) per day. With two survey teams, 10 schools can be surveyed per week and the pre-intervention surveys will be completed in all schools in 10-12 weeks. In the following term, the teams will return to conduct spot checks of attendance and to conduct classroom observations of pupil behavior and teaching methods. Two people can complete a school in one day and the assessment will take 5 weeks to complete. In the final term, the teams will return to conduct individual cognitive tests and detailed tests of literacy development. These tests will be on an individual basis and will take 4 testers 2 days to complete one school, taking around 15-20 weeks to complete.

Assessments will measure the foundational skills for literacy acquisition: specifically, letter knowledge, phonological awareness, print concepts, individual word reading, passage reading, and spelling. These foundational skills are recognized in the reading literature to be predictive of later reading acquisition in alphabetic languages. Furthermore, they are useful assessments because they are informative about a student's literacy knowledge and avoid floor effects. The assessments do not duplicate current Moe assessments. Instead, the current study will assess the literacy skills that are needed by a student if they are to meet the MoE goal that "the learners should acquire reading

skills to enable them to read and understand instructions, to read for information, and for pleasure (p. 55, Primary English Syllabus, 2002).

The method used to assess educational performance is appropriate for the local situation. People from the local community will be hired to serve as educational assessors. Since the assessments are administered individually, assessors will be trained to ensure children feel comfortable and can clarify the directions in the mother tongue. In addition, brief assessments are used in consideration of young children's attention spans.

The design of both literacy intervention and the assessment methods is the result of discussions with Ministry staff and other partners, including the Director of Primary Education, Kenya Ministry of Education, and Aga Khan Foundation and African Population and Health Research Centre (APHRC), who are developing literacy interventions for implementation in Coast Province.

The following educational assessments will be undertaken.

School attendance: Data on attendance over the year will be obtained from class attendance registers kept by teachers and which are completed daily as a routine. The registration system will be modified slightly to distinguish between absences due to illness (recorded as S) and those due to other causes (e.g. funerals, travel, home duties, recorded as O). Recording of absenteeism using the modified method will start three months prior to the intervention to establish the procedure in schools. Spot checks will be carried out for quality control. This method has worked successfully in other school health research projects in Kenya (Miguel & Kremer, 2003), although in invariably subject to reporting bias. Class attendance records will be collected at the end of each term for data entry.

School performance: In order to assess whether any improvements in attendance and attention result in increased learning at school, a test of school performance will be administered before treatment and at several subsequent points during the trial. School performance will be evaluated amongst all children in the study cohort, beginning in standard 1. We will assess all subjects but will focus particularly on the development of children's literacy and numeracy (Appendix 4 and 5). Recent evidence suggests that the Early Grade Reading Assessment (EGRA) is a cost-effective and simple means to assess children's literacy skills in the early grades (Jukes, Vagh, & Kim, 2006; RTI, 2008). Similar evaluation tools are available for early numeracy.

Assessing mechanisms for the effect on improved school performance: The impact of disease prevention on educational achievement happens through a number of different routes – more time spent in class, more time spent on-task whilst in class and improved cognitive abilities devoted to the task. The battery of behavioral assessments will be extended to capture full range of potential effects of the intervention (Appendix 6). A particular focus will be given to executive function tasks, such as sustained attention. These have been shown to be sensitive to the impact of malaria and anemia and are important for educational achievement in the early grades. Teams will also spend time in classrooms observing teacher and pupil behavior to assess how this changes in response to malaria intervention and teacher training (Jukes et al., 2006).

Tests will be conducted on the same day as, and immediately before, assessments of parasitaemia and anaemia. This will allow an analysis of the covariance between these biological indicators and ability to concentrate.

Educational covariates: Recent analyses of large school-based cluster controlled trials (Raudenbush et al., 2007) demonstrates that power can be increased considerably through the assessment of an extensive range of covariates, including teacher and school characteristics and classroom behavior. These will be assessed in the current study as part of the behavioural and educational assessment

battery. We will assess parental education and support for their children's education, teacher's levels of training, provision of textbooks, desks and other materials in the schools, and other socio-economic covariates (Appendix 7). A subset of families will be selected to conduct further interviews about how literacy development is supported in the home.

Classroom Visits:
The evaluation is interested in differences between teachers' instructional behaviors that lead to better reading outcomes. With the teacher's permission, we will video their classroom instruction while simultaneously observing. Each teacher will be observed several times with two instruments. The Stalling (Stallings, 1980) instrument codes the activities engaged in and materials used at multiple time points within one class period. The second instrument, the CLASSIC (Scanlon et al., 2003), assesses specific methods for literacy instruction. Following the lesson, the observer will meet with the teacher for about 30 minutes for a structured interview. The observations, videos, and interviews will support a more detailed analysis of how teachers' behaviors differ and help our understanding of the ways teachers can influence their pupils' reading acquisition.

Cost benefit and effectiveness analysis: The evaluation will also examine the costs, cost-benefits and cost-effectiveness of IST in improving educational and health outcomes. The costs of IST will be assessed from both a provider's and societal perspective. Both financial and opportunity costs will be evaluated using standardized methodologies **(**Drummond et al., 2005). In addition to estimating the rate of return to education, cost-benefit analysis will incorporate improved cognition and learning into the benefit streams **(**Jimenez & Patrinos, 2008). A comparison of and benefits of other education interventions in Kenya (e.g. Miguel & Kremer, 2004; Glewwe et al., 2004; Glewwe & Kremer, 2006) will also be undertaken. Cost per case of anaemia will also be assessed.

Process evaluation: This will aim to (i) investigate community acceptability of malaria intermittent screening and treatment; and (ii) document factors external to the intervention which might impact upon both its implementation and its effectiveness. Such an evaluation will help the interpretation of results and help inform future large-scale implementation of the intervention.

First, a modified stakeholder analysis approach will be adopted to identify and assess the importance of key people, or groups of people who are likely to affect the implementation and longer-term sustainability of the programme (Steckler & Linnan, 2004; Varvasovszky & Brugha, 2000). Key stakeholders are likely to include: teachers, parents, children, community leaders, local health workers and education officers as well as individuals at provincial and central levels in the Ministries of Education and Health. Stakeholders from both intervention and control schools will be included in order to obtain views on the intervention, but also the acceptability of not immediately receiving the intervention. An assessment will be made of their importance to the success and sustainability of the programme and data on their views about the programme (e.g., expectations of the intervention, experiences of the intervention, acceptability of the approach, value to individuals & communities, impact on workload) will be collected through a series of focus group discussions as well as semi-structured interviews with people identified as key informants (Bernard, 2006). Discussions and interviews will be transcribed and translated, and content analysis using Nvivo 8 (QSR International, Melbourne, Australia) will be undertaken to identify themes based on people's experience and involvement in the intervention.

Second, an analysis will be undertaken of the structural, organisational and management factors that enhance or constrain effective implementation of this and other school-based screening and treatment programs, for example, deworming and vitamin A, by staff from the Ministries of Health and Education. In addition to identifying and mapping these stakeholders through the stakeholder analysis, interviews will be conducted with purposefully selected key stakeholders in order to assess the organization and managerial capacities of the government at national and local levels (Jones et al., 2008). These data will be analysed and interpreted iteratively based on implementation and

organizational management theories (Damschroder et al. 2009) and the developed stakeholder analysis framework.

x) Laboratory procedures

Thick and thin blood smears will be stained with 2% Giemsa for 30 minutes and read by experienced laboratory technicians. Parasite densities will be calculated by counting the number of asexual parasites per 200 leukocytes (or per 500 leukocytes, if the count is <10 asexual parasites/200 leukocytes), assuming a leukocyte count of 8000/µl. A blood smear will be considered negative when the examination of 200 high power fields does not reveal asexual parasites. Thin smears will also be made for counting high parasitaemias. For quality control, all slides will be read by a second microscopist and a third reviewer will settle any discrepant readings.

Haemoglobin levels will be measured using a portable HemoCue photometer (HemoCue, Angelhom, Sweden).

Stool examinations will be conducted to determine the prevalence and intensity of intestinal nematodes, using the Kato-Katz method. Urine examinations will also be conducted to determine the prevalence and intensity of *Schistosoma haematobium,* using the urine filtration method.

xi) Sponsor

The sponsor of the study will be the London School of Hygiene and Tropical Medicine, Keppel Street, London WC1E 7HT, UK.

KEMRI, though CMRG-C, Kilifi, will take on the role of database management for active and passive case detection and monitoring of laboratory and field studies. However, SAEs will be reported to the Data Safety Monitoring Board (see below).

xii) Data Safety Monitoring Board (DSMB)

An independent committee consisting of experts in malaria, epidemiology, statistics and other appropriate disciplines has been appointed to oversee ethical and safety aspects of the study conduct. A quorum of 3 members is required at scheduled meetings.

The role of the DSMB includes the review of the implementation and progress of the study. It provides initial, regular, and closing advice on safety-related issues to the study sponsor. Its advice is based on the interpretation of study data with reference to the study protocol. The DSMB may, if deemed necessary, convene a meeting with, or request further information from the Principal Investigators and Local Safety Monitors at any stage of the study. The DSMB is empowered to suspend the enrollment to the trial and/or drug treatment on the trial pending review of potential safety issues arising in this trial or other relevant trials of the same drug product. The process will be described in study-specific SOPs. The DSMB will be informed of:

- All SAEs.
- All withdrawals of study subjects by the Principal Investigator or the parent(s)/guardian(s) of a subject due to adverse events.
- New information that may affect adversely the safety of the subjects or the conduct of the study.
- All subsequent protocol amendments, informed consent changes or revisions of other documents originally submitted for review.
- All subsequent protocol modifications (for information).

The final analysis will be conducted by the investigators, but the analysis plan will be discussed with and approved by the DSMB before it is implemented.

xiii) Local Safety Monitor (LSM)

The overall role of the Local Safety Monitors (LSM), who are experienced clinicians based in-country and based in Kilifi or Nairobi, will be to support the clinical investigators and to act as a link between the investigators and the DSMB. The LSM's role will include:

- Acting as the study volunteer's advocate.
- Promptly communicating relevant safety information to the DSMB.
- Providing advice to the investigators on whether a set of clinical circumstances in a study warrants formal notification to the DSMB.
- Conduct a site visit to investigate every report of a SAE
- Unblinding a subject if deemed necessary to allow for adequate treatment.
- Liaising closely with the chair of the DSMB throughout the course of the trial.

The relevant LSM will be informed by the investigator on an 'as received' basis of:

- All SAEs.
- All withdrawals of study subjects by the Principal Investigator or the parent(s)/ guardian(s) of a subject due to adverse events.

xiv) Adverse event monitoring

An adverse event (AE) is defined as "any untoward medical occurrence in a participant or clinical investigation participant administered a pharmaceutical product that does not necessarily have a causal relationship with this treatment" (ICH Guidelines E2A). An adverse event can further be broadly defined as any untoward deviation from baseline health which includes:

- Worsening of conditions present at the onset of the study.
- Deterioration due to the primary disease.
- Intercurrent illness.
- Events related or possibly related to concomitant medications.

A serious adverse event (SAE) is defined as an experience that results in any of the following outcomes:

- Death during the period of study follow-up.
- Life-threatening experience (one that puts a participant at immediate risk of death at the time of the event).
- Inpatient hospitalization during the period of study follow-up.
- Persistent or significant disability or incapacity.
- Specific medical or surgical intervention to prevent one of the other serious outcomes listed in the definition.

Identification of adverse events

On days 2 and 3 of treatment, study clinicians will assess children according to a standardized clinical record form (Appendix 8). Children absent from school on these days will be traced at home using information collected from parents during the consenting procedure.

Adverse events will be monitored for a further 28 days using a passive surveillance system in schools and local health centres. Travel costs will be reimbursed and treatment charges waived. Any new event, or an event present at baseline that is increasing in severity, will be considered an adverse event. Adverse events will be monitored until the event has cured or stabilized.

Reporting of adverse events

For each possible adverse event identified and graded as moderate, severe or life threatening, an adverse event report form will be completed (Appendix 9). The following information will be recorded for all adverse experiences that are reported:

- Description of event
- Date of event onset
- Date event reported
- Maximum severity of the event
- Maximum suspected relationship of the event to study medication
- Is the event serious?
- Initials of the person reporting the event
- Was the event episodic or intermittent in nature?
- Outcome
- Date event resolved

Grading of events will be based on standard guidelines (Appendix 10).

Reporting of serious adverse events
Guidelines for reporting of serious adverse events provided by the KEMRI, the London School of Hygiene & Tropical Medicine, and the data and safety monitoring board (DSMB) will be followed.

xv) Quality control

All members of the study team will be trained in the study objectives, methods of effective communication with study participants, and collection of high quality data. Study members will receive additional training specific to the tasks they will perform within the study including interviewing techniques, and clinical and laboratory measurements. A random sample of stool, urine and blood samples will be re-read by experienced technicians and any disparities corrected.

9. Data management

Data entry and storage

All questionnaires will be transferred to KIlifi for review, data entry, and storage. Data will be entered by two independent clerks, will be verified for data entry errors, and corrected from the original questionnaire using customized software (Microsoft Visual FoxPro® 6.0) and verified for accuracy. Study records will be stored securely in KEMRI facilities.

Data analysis

All analyses will be on the basis of intention-to-treat. To assess the success of randomization baseline characteristics between the four randomization groups will be compared. To assess the impact of the interventions, methods appropriate for cluster-randomized trials will be used (Hayes & Bennett, 1999). The prevalence, or mean, in each school will be calculated and the unadjusted risk ratio (RR), or mean difference (intervention-control), estimated in each stratum. An overall estimate of the effect of IST will be obtained by taking a weighted average of the stratum-specific estimates, the weights proportional to the number of schools per stratum, and 95% confidence intervals will be adjusted for observed between-school variance. Formal hypothesis testing will be undertaken using stratified unpaired t-tests. The impact of each intervention will be estimated for education outcomes and health outcomes in separate multiple regression models. An interaction term will be included to identify any synergistic effects between the two interventions. Statistical analysis will be carried out using STATA® software, version 10.0. (Stata Corporation, Texas, USA).

10. Time Frame

**2008**											Nov	Dec
Staff recruitment and training											■	■
Development and piloting of education assessment tools											■	■
**2009**	Jan	Feb	Mar	Apr	May	Jun	Jul	Aug	Sep	Oct	Nov	Dec
Development and piloting of education assessment tools	■											
Teacher training workshop	■											
Baseline health and education surveys		■	■									
IST				■								
Instruction intervention				■	■	■						
Assessment of class behaviour						■						
**2010**	Jan	Feb	Mar	Apr	May	Jun	Jul	Aug	Sep	Oct	Nov	Dec
First follow-up health and education surveys		■	■									
IST				■								
Instruction intervention				■	■	■						
Assessment of class behaviour						■						
First follow-up health and education surveys												■

11. Ethical considerations

Informed consent process

Prior to the onset of the study, parent-teacher association meetings will be held with the parents/guardians of children enrolled in standards 1-5 to describe the purpose of the study, the procedures to be followed, and the risks and benefits of participation. Information sheets (Appendix 11) will be provided to the parents or guardians for their review. For those parents who not attend these meetings, follow-up will be made through community leaders and household visits.

The parents or guardians will be asked to sign consent for their child (if aged under 18 years) to participate in the research study (Appendix 11). If the child is aged 18 years or over, is married or is a mother (so-called emancipated minor by Kenyan law), they will be asked to provide consent themselves. If a parent, guardian or older child is unable to read or write, his/her fingerprint will be used in substitute for a signature, and a signature from a witness to the informed consent discussion will be obtained. Parents and guardians will have the chance to ask questions and will be informed that participation of their child(ren) in the study is completely voluntary and that they may withdraw from the study at any time.

Information about all children, including age, gender, and any history of known allergies or adverse reactions to study medications, will be obtained from parents/guardians and captured on an initial screening form (Appendix 2). Details about the location of the students' homes will also be obtained from the parent/guardians to facilitate tracing in the event of absence from school on subsequent follow-up visits. A record will be made of those children for whom consent is provided and will be cross-checked against school registers.

Written consent to participate in the stakeholder interviews and focus groups discussions will be sought.

Verbal assent to participate in the study will also be obtained from the child at the time of screening (Appendix 11).

Training for those involved in administering consent

All fieldworkers will undergo training prior to both studies. Training will educate fieldworkers on the purpose of the study, the importance of consent and how to administer both the consent forms and questionnaires. While in the field, fieldworkers will have continuous contact through the use of a mobile phone with the team leaders in case of any queries.

Community engagement
Prior to the onset of the study, meetings will also be held with officials from the District Education Office, the District Health Management Team and District Commissioner's office to sensitize them about the study and plans for recruitment and follow-up. Similar meeting will also be held with official at the national level in Nairobi.

Upon completion of the study, a two page 'Research Brief' will be developed, similar to that developed for the Bondo study. In addition, a one-day meeting with headteachers of participating schools, district health and education staff will be held, where the results of the study will be presented and discussed. Headteachers will be asked to distribute the Research Brief to interested parents. At the national level, results will be disseminated primarily to the Division of Malaria Control (Ministry of Public Health and Sanitation) and the School Health and Nutrition Programme (Ministry of Education). Finally, a half-day workshop will be convened in Nairobi by the World Bank where the implications of the results will be discussed in light of current school health plans.

Ethical approval
Ethical approval for this study will be obtained from KEMRI National Ethical Committee and the London School of Hygiene and Tropical Medicine before commencement of the study.

Benefits
This study has been designed to address several areas of major public health and educational significance for Kenyan schoolchildren. If we are able to show that malaria treatment improves educational outcomes, millions of African schoolchildren living in malaria endemic regions may benefit.

Children randomised to treatment intervention arms will benefit from free medical treatment for asymptomatic malaria infections. Rapid malaria tests will be performed for those who have a history of fever or raised temperature so that those with malaria parasites can immediately receive treatment, or referred to the nearest health facility, instead of waiting for the slides to be read.

Risks
Parents/guardians and children will be informed of all potential risks.

This study involves collection of finger-prick blood samples which may lead to minor temporary discomfort or pain for children. Precautions will be taken to avoid bleeding by immediate application of sterilized cotton wool and pressure at the prick site. Risks of infection will be minimized by using disposable lancets, one for each child to avoid cross contamination/transmission of infectious agents. Stool and urine sample collection may be embarrassing for the children, but this discomfort will be minimized by guaranteeing children's privacy during stool collection.

The study involves specific risks from the from the artemisinin-based combination therapy (ACT) drugs. ACTs are remarkably well tolerated in humans, with no serious adverse events or significant toxicity reported.

Confidentiality
Participants, parents and guardians will be informed that participation in a research study may involve a loss of privacy. All records will be kept as confidential as possible. Participants will be

identified primarily by their study number and patient names will not be entered into the computerized database. No individual identities will be used in any reports or publications resulting from the study.

12. Expected Application of Results

The results will be disseminated primarily to the Division of Malaria Control (Ministry of Public Health and Sanitation) and the School Health and Nutrition Programme (Ministry of Education), as well as to the District Health Management Team and District Education Office, and other interested stakeholders. This study form part of the evidence-base required by the government of Kenya to inform the decision as to the appropriate malaria control strategy in schools. For example, it will be important to know whether both health and education intervention is required to improve educational outcomes. The evidence may also be useful to other African countries thinking of developing similar interventions and to national and international organizations who may be willing to fund such interventions at a national level. It is also anticipated that the results will be presented at appropriate national and international scientific meetings and several papers will be written and submitted to peer-reviewed scientific journals for publication.

13. References

Adams, M. J. (1990). *Beginning to read: Thinking and learning about print*. Cambridge, MA: MIT Press.

August, D. & Shanahan, T. (Eds.) (2006). *Developing literacy in second-language learners: Report of the National Literacy Panel on Language-Minority Children and Youth.* Mahwah, NJ: Lawrence Erlbaum Associates.

Bernard HR (2006) Research methods in anthropology: qualitative and quantitative approaches. Oxford, UK: AltaMira Press.

Blair, C., & Razza, R. P. (2007). Relating effortful control, executive function, and false belief understanding to emerging math and literacy ability in kindergarten. *Child Development, 78*(2), 647-663.

Brooker, S., Guyatt, H., Omumbo, J., Shretta, R., Drake, L., & Ouma, J. (2000). Situation analysis of malaria in school-aged children in Kenya - what can be done? *Parasitology Today, 16*(5), 183-186.

Clarke, S. E., Brooker, S., Njagi, J. K., Njau, E., Estambale, B., Muchiri, E., et al. (2004). Malaria morbidity among school children living in two areas of contrasting transmission in western Kenya. *Am J Trop Med Hyg, 71*(6), 732-738.

Clarke SE, Jukes MCH, Njagi K, Khasakhala L, Cundill B, Otido J, Crudder C, Estambale B, Brooker S: Health and educational impact of school-based intermittent preventive treatment in Kenya: a placebo cluster-randomised controlled trial. *Lancet* 372, 127-138.

Commeyras, M. & Inyega H. N. (2007). An integrative review of teaching reading in Kenyan primary schools *Reading Research Quarterly, 42,* 258-281.

Damschroder LJ, Aron DC, Keith RE, Kirsh SR, Alexander JA, Lowery JC (2009). Fostering implementation of health services research findings into practice: a consolidated framework for advancing implementation science. *Implementation Science*, 4:50.

Denis, M. B., Davis, T. M., Hewitt, S., Incardona, S., Nimol, K., Fandeur, T., et al. (2002). Efficacy and safety of dihydroartemisinin-piperaquine (Artekin) in Cambodian children and adults with uncomplicated falciparum malaria. *Clin Infect Dis, 35*(12), 1469-1476.

Drummond MF, Sculpher MJ, Torrance GW, O'Brien BJ, Stoddart GL: *Methods for the economic evaluation of health care programmes. Third edition.* Oxford: Oxford University Press; 2005.

Duflo E & Kremer M. Use of Randomization in the Evaluation of Development Effectiveness. In *Evaluating Development Effectiveness* (World Bank Series on Evaluation and Development,

Vol 7). Edited by Osvaldo Feinstein, Gregory K. Ingram and George K. Pitman. New Brunswick, NJ: Transaction Publishers; 2004: 205-232.
Fernando, D., Wickremasinghe, R., Mendis, K. N., & Wickremasinghe, A. R. (2003a). Cognitive performance at school entry of children living in malaria-endemic areas of Sri Lanka. *Transactions of the Royal Society of Tropical Medicine and Hygiene, 97*(2), 161-165.
Fernando, S. D., Gunawardena, D. M., Bandara, M., De Silva, D., Carter, R., Mendis, K. N., et al. (2003b). The impact of repeated malaria attacks on the school performance of children. *American Journal of Tropical Medicine and Hygiene, 69*(6), 582-588.
Geerligs, P. D., Brabin, B. J., & Eggelte, T. A. (2003). Analysis of the effects of malaria chemoprophylaxis in children on haematological responses, morbidity and mortality. *Bull World Health Organ, 81*(3), 205-216.
Glewwe P, Kremer M., Moulin S, Zitzewitz E. Retrospective vs. prospective analyses of school inputs: The case of flip charts in Kenya." *Journal of Development Economics* 5004, 74: 251–268.
Glewwe P, Kremer M. Schools, Teachers, and Education Outcomes in Developing Countries," in E.A. Hanushek and F. Welch, eds., *Handbook of the Economics of Education,* Volume 2. Elsevier, 2006.
Hayes RJ, Bennett S (1999).Simple sample size calculations for cluster-randomized trials. *Int J Epidemiol* 28: 319–326
Holding, P. A., & Snow, R. W. (2001). Impact of Plasmodium falciparum malaria on performance and learning: review of the evidence. *Am J Trop Med Hyg, 64*(1-2 Suppl), 68-75.
Jimenez E, Patrinos HA. *Can Cost-Benefit Analysis Guide Education Policy in Developing Countries?* Policy Research Working Paper 4568. Washington DC: World Bank, 2008
Jimerson, S., Egeland, B., & Teo, A. (1999). A longitudinal study of achievement trajectories: Factors associated with change.
Jones C, Abeku TA, Rapuoda B, Okia M, Cox J (2008). District-based malaria epidemic early warning systems in East Africa: perceptions of acceptability and usefulness among key staff at health facility, district and central levels. *Social Science and Medicine*, 67(2):292-300.
Jukes, M. C. H., Vagh, S. B., & Kim, Y. S. (2006). *Development of Assessments of Reading Ability and Classroom Behavior*: World Bank.
Karunajeewa, H., Lim, C., Hung, T. Y., Ilett, K. F., Denis, M. B., Socheat, D., et al. (2004). Safety evaluation of fixed combination piperaquine plus dihydroartemisinin (Artekin) in Cambodian children and adults with malaria. *Br J Clin Pharmacol, 57*(1), 93-99.
Kurtzhals, J. A., Addae, M. M., Akanmori, B. D., Dunyo, S., Koram, K. A., Appawu, M. A., et al. (1999). Anaemia caused by asymptomatic Plasmodium falciparum infection in semi-immune African schoolchildren. *Trans R Soc Trop Med Hyg, 93*(6), 623-627.
Lalloo, D., Olukoya, P, Olliaro, P. (2006). Malaria in adolescence: burden of disease, consequneces and oppurtunities for intervention. *Lancet Infectious diseases, 6,* 780-793.
Leigton, C. & Foster, R (1993). *Economic impacts of malaria in Kenya and Nigeria.* Major Applied Research Paper no 6, HFS project, Abt Associates, Bethesda.
Miguel, E., & Kremer, M. (2004). Worms: Identifying impacts on education and health in the presence of treatment externalities. *Econometrica,, 72*(1), 159-217.
Ministry of Education Science and Technology (MOEST) (2001). *Teaching and learning English in the primary classroom: English module.* Nairobi: Jomo Kenyatta Foundation.
Muthwii, M.J. (2004). Language planning and literacy in Kenya: Living with unresolved paradoxes. *Current Issues in Language Planning, 5,* 34—50.
National Institute of Child Health and Human Development (NICHHD) (2001). *Report of the National Reading Panel. Teaching children to read: An evidence-based assessment of the scientific research literature on reading and its implications for reading instruction* (NIH Publication No. 00-4769). Washington, DC: U.S. Government Printing Office.
Nzomo, J., Kariuki, M., & Guantai, L. (2001). *The quality of primary education in Kenya: Some suggestions based on a survey of schools.* Paris: International Institute for Educational Planning/United Nations Educational, Scientific and Cultural Organization.

Olliaro P, Nevill C, LeBras J, Ringwald P, Mussano P, Garner P, Brausseur P (1996). Systematic review of amodiaquine treatment in uncomplicated malaria. *Lancet* **348:**1196-1201.

Phillips-Howard PA, West LJ (1990). Serious adverse drug reactions to pyrimethamine-sulphadoxine, pyrimethamine-dapsone and to amodiaquine in Britain. *Journal of the Royal Society of Medicine* **83**(2)**:**82-85.

Raudenbush, S. W., Martinez, A., & Spybrook, J. (2007). Strategies for improving precision in group-randomized experiments. *Educational Evaluation and Policy Analysis, 29*(1), 5-29.

RTI. (2008). *Early Grade Reading Assessment (EGRA). 2nd Workshop.*, Washington, DC.

Scanlon DM, Gelzheiser L, Fanuele D, Sweeney J & Newcomer L (2003). Classroom Language Arts Systematic Sampling and Instructional Coding (CLASSIC). Unpublished manuscript, Child Research and Study Center, The University at Albany.

Snow, C. E., Burns, M. S., & Griffin, P. (Eds.). (1998). *Preventing Reading Difficulties in Young Children*. Washington, DC: National Academy Press.

Snow RW, Craig MH, Newton CRJC, Steketee RW (2003). The public health burden of *Plasmodium falciparum* malaria in Africa: Deriving the number. Working Paper No. 11, Disease Control Priorities Project. Bethesda, Maryland: Fogarty International Center, National Institutes of Health.

Stallings J (1980). Allocated academic learning time revisited, or beyond time on task. *Educational Researcher* 9, 11-16.

Steckler L, Linnan A (2004). Process evaluation for public health interventions and research San Francisco: Jossey-Bass.

Sturchler D, Mittelholzer ML, Kerr L (1993). How frequent are notified severe cutaneous adverse reactions to Fansidar? *Drug Safety* **8**(2)**:**160-68.

Taylor WR, White NJ (2004) Antimalarial drug toxicity: A review. *Drug Safety* 27:25–61.

Temperley M, Mueller D, Njagi K, Akhwale W, Clarke SE, Jukes MCH, Estambale BBA & Brooker S (2008) Costs and cost-effectiveness of delivering intermittent preventive treatment for malaria through schools in western Kenya. *Malaria Journal* 7, 196.

Varvasovszky Z, Brugha R (2000): A stakeholder analysis. *Health Policy and Planning*, 15(3):338-345.

14. Budget ($1= 76.3 Ksh)

**Item**	**Amount (US$)**	**Amount (Kshs)**
a) Personnel salaries and benefits	259,000	19,761,700
b) Patient Costs	22,500	1,716,750
c) Equipment	32,450	2,475,935
d) Supplies	16,750	7,382,025
e) Travel and accommodation	16,000	1,220,800
f) Transportation	86,450	6,596,135
g) Operating expenses	260,500	19,876,150
h) Animals	Not applicable	Not applicable
i) Consultancy fees	Not applicable	Not applicable
j) Contingency funds	75,000	5,722,500
k) Institutional administrative overheads	Not applicable	Not applicable
**Total**	**768,650**	**58,647,995**

15. Justification of Budget

Personal salaries and benefits. This includes salaries and benefits of field coordinators, fieldworkers and technicians.

Patient costs. This includes anti-malarials and placebos for all children as well as albendazole, prazquantel, Co-artem and ferrous sulphate, as required.

Equipment. This is estimated for the purchase of motorcycles large equipment including microscopes and Hemocue machines.

Supplies. This is the estimated cost of all the non-field work supplies required for the study at the KEMRI-Wellcome unit in Nairobi. It includes photocopying, stationary, communication and printing over the survey period. The estimates are based on routine costs charged at the unit.

Travel and transportation. During the study preparation an investigator will need to meet with local officials and school head teachers. Travel and accommodation costs for these trips have been factored in here.

The education component of the study involves modifying the way Standard 1 and 2 classroom teachers develop their students' literacy skills. To do this, the intervention includes an initial residential teacher training over a four-day period followed by ongoing monitoring and support. To ensure that all of the school's personnel are informed, the Head Teachers and the Senior Teachers will also be invited to the training. This way, they will be available to support the classroom teachers during the implementation of the intervention. The literacy intervention will be implemented in 50 schools involving at least three teachers from each.

The study has a longitudinal design to investigate the interaction of the malaria intervention and the literacy intervention. To understand the effects of the interventions we will measure students' education achievement at the onset and end of the interventions. Student achievement in 100 schools will be measured. While the surveys are going on, a supervisor, three laboratory technicians, one laboratory technician, one interviewer, one nurse plus one driver will need to travel to the field sites to undertake school surveys. All costs for travel and accommodation have been estimated using standard KEMRI mileage and per diem charges.

*Operating costs***.** This includes all costs required to carry out the field studies and include laboratory materials, supplies, reagents and disposables. Training workshops will be organized by ESACIPAC to train all the technicians at the DVBD in Kwale hospitals on parasite microscopy. Also included are laboratory quality control costs.
