# Supplementary material for: Impact of Intermittent Screening and Treatment for Malaria among School Children in Kenya: A Cluster Randomised Trial
Source: PLoS Med. 2014 Jan 28;11(1):e1001594. doi: 10.1371/journal.pmed.1001594 (PMC3904819; doi:10.1371/journal.pmed.1001594)
Supplement: Table S1 — Baseline measures for 5,233 study children with missing 12-months follow-up health data versus those not missing 12-months follow-up health data across both the control and IST intervention groups. (DOC) [file pmed.1001594.s006.doc]

**Table S1. Baseline measures for 5233 study children with missing 12 months follow-up health data vs. those not missing 12 months follow-up health data across both the control and IST intervention groups.**

| Characteristic; n (%) ^a^ |  | **CONTROL GROUP** | | **INTERVENTION GROUP** | |
| --- | --- | --- | --- | --- | --- |
|  |  | Missing outcome data | Outcome data available | Missing outcome data | Outcome data available |
| Child characteristics |  | N=375 | N=2148 | N=412 | N=2298 |
| **Age** | Mean (sd) | 10.4 (3.1) | 10.1 (2.8) | 10.6 (3.1) | 10.3 (2.8) |
|  | 5-9 | 155 (41.3) | 886 (41.2) | 155 (37.6) | 914 (39.8) |
|  | 10-12 | 107 (28.5) | 770 (35.9) | 120 (29.1) | 805 (35.0) |
|  | 13-20 | 113 (30.1) | 492 (22.9) | 137 (33.3) | 579 (25.2) |
| **Sex** | Male | 193 (51.5) | 1064 (49.5) | 208 (50.5) | 1111 (48.3) |
| **Child sleeps under net** | Usually | 229 (63.6) | 1439 (67.9) | 238 (60.1) | 1444 (63.7) |
|  | Last night | 223 (97.4) | 1383 (96.1) | 225 (94.5) | 1384 (95.8) |
| **Nutritional Status** | Underweight | 42 (30.7) | 224 (26.4) | 26 (22.6) | 205 (24.1) |
|  | Stunted | 80 (24.1) | 520 (25.3) | 72 (22.4) | 540 (25.2) |
|  | Thin | 64 (19.3) | 418 (20.4) | 47 (14.6) | 403 (18.8) |
| Household characteristics |  |  |  |  |  |
| **Parental Education** | No schooling | 101 (28.2) | 625 (29.6) | 158 (39.6) | 767 (33.8) |
|  | Primary schooling | 180 (50.3) | 1112 (52.6) | 196 (49.1) | 1185 (52.2) |
|  | Secondary schooling | 59 (16.5) | 294 (13.9) | 30 (7.5) | 248 (10.9) |
|  | Higher education | 18 (5.0) | 84 (4.0) | 15 (3.8) | 68 (3.0) |
| **Socioeconomic status** | Poorest | 67 (18.6) | 373 (17.6) | 98 (24.5) | 557 (24.4) |
|  | Poor | 84 (23.3) | 399 (18.8) | 88 (22.0) | 476 (20.9) |
|  | Median | 63 (17.5) | 402 (18.9) | 84 (21.0) | 411 (18.0) |
|  | Less poor | 60 (16.7) | 464 (21.8) | 72 (18.0) | 437 (19.2) |
|  | Least poor | 86 (23.9) | 486 (22.9) | 58 (14.5) | 400 (17.5) |
| **Household size** | 1-5 | 122 (33.9) | 575 (27.1) | 117 (29.5) | 586 (25.8) |
|  | 6-9 | 193 (53.6) | 1251 (59.0) | 211 (53.3) | 1369 (60.3) |
|  | 10-31 | 45 (12.5) | 293 (13.8) | 68 (17.2) | 314 (13.8) |
| Study endpoints-baseline |  | Class 1 N=183  Class 5 N=192 | Class 1 N=1039  Class 5 N=1109 | Class 1 N=191  Class 5 N=221 | Class 1 N=1126  Class 5 N=1172 |
| **Anaemia prevalence** | Age-sex specific | 144 (44.4) | 929 (45.3) | 128 (41.6) | 986 (46.0) |
|  | Severe (<70g/L) | 2 (0.6) | 12 (0.6) | 0 (0.0) | 14 (0.7) |
|  | Moderate (70-89 g/L) | 10 (3.1) | 33 (1.6) | 7 (2.3) | 48 (2.2) |
|  | Mild (90-109 g/L) | 66 (20.4) | 464 (22.6) | 55 (17.9) | 463 (21.6) |
|  | None (≥110 g/L) | 246 (75.9) | 1540 (75.2) | 246 (79.9) | 1618 (75.5) |
| **Haemoglobin (g/L)** | Mean (sd) | 117.7 (13.6) | 117.3 (12.9) | 118.9 (13.3) | 117.3 (13.7) |
| ***P.falciparum* prevalence** ^b^ |  | - - | - - | 26 (8.6) | 285 (13.6) |
| **Class 1** ^c^ |  |  |  |  |  |
| Score: 0-20 | Sustained attention ^d^ | 11.9 (6.7) [0, 20] | 11.9 (6.7) [0, 20] | 11.8 (6.6) [0, 20] | 12.2 (6.6) [0, 20] |
| Score: 0-20 | Spelling | 8.0 (4.2) [0, 19] | 8.7 (4.5) [0, 19] | 7.4 (4.5) [0, 19] | 7.7 (4.4) [0, 20] |
| Score: 0-30 | Arithmetic | 2.4 (2.3) [0, 12] | 2.6 (2.4) [0, 17] | 2.3 (2.6) [0, 13] | 2.6 (2.5) [0, 15] |
| **Class 5**  ^c^ |  |  |  |  |  |
| Score: 0-20 | Sustained attention ^d^ | 9.9 (6.1) [0, 20] | 9.9 (6.0) [0, 20] | 9.6 (5.7) [0, 20] | 10.6 (5.7) [0, 20] |
| Score: 0-78 | Spelling | 24.0 (11.6) [0, 51] | 28.6 (11.7) [0, 63] | 24.2 (11.1) [0, 56] | 26.1 (11.2) [0, 59] |
| Score: 0-38 | Arithmetic | 28.6 (6.1) [5, 38] | 29.5 (5.5) [0, 38] | 27.2 (7.0) [1, 38] | 28.8 (5.5) [0, 38] |

^a^ % of non-missing children in each study group presented for categorised data. For continuous data mean(sd) [min,max] is presented;

^b^ Not measured at baseline in the control group;

^c^ Presented as mean(sd) [min,max]

^d^ In class 1 sustained attention was measured by the “pencil tap test” and in class 5 sustained attention was measured by the “two digit code transmission test”.
