# Supplementary material for: Impact of Intermittent Screening and Treatment for Malaria among School Children in Kenya: A Cluster Randomised Trial
Source: PLoS Med. 2014 Jan 28;11(1):e1001594. doi: 10.1371/journal.pmed.1001594 (PMC3904819; doi:10.1371/journal.pmed.1001594)
Supplement: Table S2 — Baseline measures for 5,233 study children with missing 24-months follow-up health data versus those not missing 24-months follow-up health data across both the control and IST intervention groups. (DOC) [file pmed.1001594.s007.doc]

**Table S2. Baseline measures for 5233 study children with missing 24 months follow-up health data vs. those not missing 24 months follow-up health data across both the control and IST intervention groups.**

| Characteristic; n (%) ^a^ |  | **CONTROL GROUP** | | **INTERVENTION GROUP** | |
| --- | --- | --- | --- | --- | --- |
|  |  | Missing outcome data | Outcome data available | Missing outcome data | Outcome data available |
| Child characteristics |  | N=496 | N=2027 | N=536 | N=2174 |
| **Age** | Mean (sd) | 10.5 (3.1) | 10.0 (2.8) | 10.9 (3.1) | 10.2 (2.7) |
|  | 5-9 | 196 (39.5) | 845 (41.7) | 184 (34.3) | 885 (40.7) |
|  | 10-12 | 140 (28.2) | 737 (36.4) | 149 (27.8) | 776 (35.7) |
|  | 13-20 | 160 (32.3) | 445 (22.0) | 203 (37.9) | 513 (23.6) |
| **Sex** | Male | 240 (48.4) | 1017 (50.2) | 248 (46.3) | 1071 (49.3) |
| **Child sleeps under net** | Usually | 308 (64.4) | 1360 (68.0) | 324 (62.4) | 1358 (63.3) |
|  | Last night | 298 (96.8) | 1308 (96.2) | 310 (95.7) | 1299 (95.7) |
| **Nutritional Status** | Underweight | 50 (28.6) | 216 (26.7) | 27 (18.7) | 204 (24.8) |
|  | Stunted | 102 (23.0) | 498 (25.7) | 106 (24.3) | 506 (25.0) |
|  | Thin | 76 (17.1) | 406 (20.9) | 66 (15.1) | 384 (19.0) |
| Household characteristics |  |  |  |  |  |
| **Parental Education** | No schooling | 147 (30.8) | 579 (29.0) | 203 (39.0) | 722 (33.6) |
|  | Primary schooling | 237 (49.7) | 1055 (52.9) | 257 (49.4) | 1124 (52.4) |
|  | Secondary schooling | 75 (15.7) | 278 (13.9) | 42 (8.1) | 236 (11.0) |
|  | Higher education | 18 (3.8) | 84 (4.2) | 18 (3.5) | 65 (3.0) |
| **Socioeconomic status** | Poorest | 95 (19.8) | 345 (17.2) | 124 (23.8) | 531 (24.6) |
|  | Poor | 105 (21.9) | 378 (18.9) | 115 (22.0) | 449 (20.8) |
|  | Median | 87 (18.2) | 378 (18.9) | 99 (19.0) | 396 (18.3) |
|  | Less poor | 73 (15.2) | 451 (22.5) | 105 (20.1) | 404 (18.7) |
|  | Least poor | 119 (24.8) | 453 (22.6) | 79 (15.1) | 379 (17.6) |
| **Household size** | 1-5 | 158 (33.1) | 539 (26.9) | 144 (27.7) | 559 (26.0) |
|  | 6-9 | 262 (54.8) | 1182 (59.1) | 298 (57.4) | 1282 (59.7) |
|  | 10-31 | 58 (12.1) | 280 (14.0) | 77 (14.8) | 305 (14.2) |
| Study endpoints-baseline |  | Class 1 N=230  Class 5 N=266 | Class 1 N=992  Class 5 N=1035 | Class 1 N=226  Class 5 N=310 | Class 1 N=1091  Class 5 N=1083 |
| **Anaemia prevalence** | Age-sex specific | 206 (47.0) | 867 (44.8) | 194 (45.9) | 920 (45.4) |
|  | Severe (<70g/L) | 2 (0.5) | 12 (0.6) | 1 (0.2) | 13 (0.6) |
|  | Moderate (70-89 g/L) | 8 (1.8) | 35 (1.8) | 9 (2.1) | 46 (2.3) |
|  | Mild (90-109 g/L) | 98 (22.4) | 432 (22.3) | 83 (19.6) | 435 (21.4) |
|  | None (≥110 g/L) | 330 (75.3) | 1456 (75.2) | 330 (78.0) | 1534 (75.6) |
| **Haemoglobin (g/L)** | Mean (sd) | 117.3 (13.3) | 117.3 (12.9) | 118.5 (13.6) | 117.3 (13.7) |
| ***P.falciparum* prevalence** ^b^ |  | - - | - | 37 (8.9) | 274 (13.8) |
| **Class 1** ^c^ |  |  |  |  |  |
| Score: 0-20 | Sustained attention ^d^ | 11.6 (6.7) [0, 20] | 11.9 (6.7) [0, 20] | 11.6 (6.8) [0, 20] | 12.3 (6.5) [0, 20] |
| Score: 0-20 | Spelling | 8.5 (4.1) [0, 19] | 8.6 (4.6) [0, 19] | 7.7 (4.7) [0, 19] | 7.6 (4.4) [0, 20] |
| Score: 0-30 | Arithmetic | 2.6 (2.3) [0, 12] | 2.6 (2.4) [0, 17] | 2.6 (2.8) [0, 15] | 2.6 (2.4) [0, 12] |
| **Class 5**  ^c^ |  |  |  |  |  |
| Score: 0-20 | Sustained attention ^d^ | 9.8 (6.1) [0, 20] | 9.9 (6.0) [0, 20] | 9.4 (5.5) [0, 20] | 10.7 (5.7) [0, 20] |
| Score: 0-78 | Spelling | 24.2 (11.4) [0, 52] | 28.9 (11.7) [0, 63] | 22.5 (10.7) [1, 51] | 26.7 (11.1) [1, 59] |
| Score: 0-38 | Arithmetic | 28.6 (6.2) [4, 38] | 29.6 (5.4) [0, 38] | 27.3 (6.4) [3, 38] | 28.8 (5.6) [0, 38] |

^a^ % of non-missing children in each study group presented for categorised data, where data is continuous mean(sd) is presented.

^b^ Not measured at baseline in the control group;

^c^ Presented as mean(sd) [min,max]

^d^ In class 1 sustained attention was measured by the “pencil tap test” and in class 5 sustained attention was measured by the “two digit code transmission test”.
