# Supplementary material for: Impact of Intermittent Screening and Treatment for Malaria among School Children in Kenya: A Cluster Randomised Trial
Source: PLoS Med. 2014 Jan 28;11(1):e1001594. doi: 10.1371/journal.pmed.1001594 (PMC3904819; doi:10.1371/journal.pmed.1001594)
Supplement: Table S3 — Results from missing data analysis for anaemia. Effect of the IST intervention at 12- and 24-months follow-up on the primary health outcome of anaemia for study children combined using a longitudinal, random effects regression modeling approach. Results presented (i) for all children with either 12- or 24-months follow-up measurements of the outcome (unadjusted), (ii) for those with baseline measurements of the outcome and accounting for age, sex, and stratification effects as the primary pre-specified analysis, and (iii) for those additionally with baseline measures of parental education, SES, and baseline educational level (measured by baseline spelling) as further predictors of missingness. (DOC) [file pmed.1001594.s008.doc]

**Table S3. Results from missing data analysis for anaemia.** Effect of the IST intervention at 12 and 24 months follow-up on the primary health outcome of anaemia for study children combined using a longitudinal, random effects regression modeling approach. Results presented (i) for all children with either 12 or 24 months follow-up measurements of the outcome (unadjusted), (ii) for those with baseline measurements of the outcome and accounting for age, sex and stratification effects as the primary pre-specified analysis, and (iii) for those additionally with baseline measures of parental education, SES and baseline educational level (measured by baseline spelling) as further predictors of missingness.

| **Prevalence of anaemia^a^** | **Control**  **(50 schools)** | | **Intervention**  **(51 schools)** | | **Odds ratio^c^**  **(95% CI)** | **p-value** ^d^ | **ICC (95% CI)** | |
| --- | --- | --- | --- | --- | --- | --- | --- | --- |
|  |  | **n (%)^b^** |  | **n (%)^b^** |  |  | School | Child |
| **Unadjusted** |  |  |  |  |  |  |  |  |
| 12-month | 2146 | 837 (39.0%) | 2297 | 920 (40.1%) | 1.08 (0.74,1.43) | 0.758 | 0.07 (0.05,0.10) | 0.50 (0.45,0.54) |
| 24-month | 2027 | 809 (39.9%) | 2173 | 910 (41.9%) | 1.12 (0.77,1.48) |
| **Adjusted** |  |  |  |  |  |  |  |  |
| 12-month | 2048 | 788 (38.5%) | 2142 | 858 (40.1%) | 1.09 (0.79,1.40) | 0.890 | 0.06 (0.04,0.08) | 0.38 (0.33,0.43) |
| 24-month | 1935 | 765 (39.5%) | 2027 | 842 (41.5%) | 1.11 (0.80,1.42) |
| **Adjusted for predictors of missingness** | | |  |  |  |  |  |  |
| 12-month | 1998 | 768 (38.4%) | 2083 | 832 (39.9%) | 1.05 (0.77,1.34) | 0.789 | 0.05 (0.03,0.07) | 0.34 (0.32,0.42) |
| 24-month | 1889 | 747 (39.5%) | 1969 | 820 (41.7%) | 1.09 (0.79,1.38) |

**^a^** Age-sex specific anaemia was defined using age and sex corrected WHO thresholds of haemoglobin concentration: <110g/l in children under 5 years; <115g/l in children 5 to 11 years; <120g/l in females 12 years and over and males 12 to 14.99 years old; and <130g/l in males ≥ 15 years. All female adolescents are assumed to not be pregnant

**^b^** Number and percentage with outcome

^c^ Odds ratios (intervention/control) presented for anaemia are obtained from random effects logistic regression analysis accounting for school-level clustering and repeated measures of children for the comparison of the intervention effect at 12 months to 24 months

^d^ p-value for the comparison of the intervention effect at 12 months to 24 months

**Unadjusted**: All children with outcome measures, not adjusted for any baseline or study design characteristics.

**Adjusted**: for baseline age, sex, school mean exam score and literacy group (to account for stratification) and baseline measure of the outcome, where available.

**Adjusted for predictors of missingness**: for baseline age, sex, school mean exam score and literacy group (to account for stratification) and baseline measure of the outcome, where available. Additionally adjusted for parental education, SES and baseline educational level as measured by baseline spelling score (standardized by subtracting year-group baseline mean and scaled by year-group sd).
