# Supplementary material for: Impact of Intermittent Screening and Treatment for Malaria among School Children in Kenya: A Cluster Randomised Trial
Source: PLoS Med. 2014 Jan 28;11(1):e1001594. doi: 10.1371/journal.pmed.1001594 (PMC3904819; doi:10.1371/journal.pmed.1001594)
Supplement: Table S4 — Baseline measures for study children with missing 9-months follow-up education data versus those not missing 9-months follow-up education data across both the control and intervention groups. (DOC) [file pmed.1001594.s009.doc]

**Table S4. Baseline measures for study children with missing 9 months follow-up education data vs. those not missing 9 months follow-up education data across both the control and intervention groups.**

| Characteristic; n (%) ^a^ |  | **CONTROL GROUP** | | **INTERVENTION GROUP** | |
| --- | --- | --- | --- | --- | --- |
|  |  | Missing outcome data | Outcome data available | Missing outcome data | Outcome data available |
| Child characteristics |  | N=265 | N=2258 | N=312 | N=2398 |
| **Age** | Mean (sd) | 10.0 (3.2) | 10.1 (2.8) | 10.5 (3.1) | 10.3 (2.8) |
|  | 5-9 | 125 (47.2) | 916 (40.6) | 121 (38.8) | 948 (39.5) |
|  | 10-12 | 71 (26.8) | 806 (35.7) | 91 (29.2) | 834 (34.8) |
|  | 13-20 | 68 (26.0) | 536 (23.7) | 100 (32.1) | 616 (25.7) |
| **Sex** | Male | 134 (50.6) | 1123 (49.7) | 157 (50.3) | 1162 (48.5) |
| **Child sleeps under net** | Usually | 167 (66.8) | 1501 (67.3) | 174 (58.6) | 1508 (63.7) |
|  | Last night | 164 (98.2) | 1442 (96.1) | 169 (97.1) | 1440 (95.5) |
| **Nutritional Status** | Underweight | 38 (34.9) | 228 (26.0) | 17 (19.5) | 214 (24.3) |
|  | Stunted | 55 (24.6) | 545 (25.2) | 44 (19.2) | 568 (25.4) |
|  | Thin | 48 (21.4) | 434 (20.1) | 39 (17.0) | 411 (18.4) |
| Household characteristics |  |  |  |  |  |
| **Parental Education** | No schooling | 81 (32.5) | 645 (29.0) | 127 (42.8) | 798 (33.7) |
|  | Primary schooling | 128 (51.4) | 1164 (52.3) | 141 (47.5) | 1240 (52.3) |
|  | Secondary schooling | 30 (12.0) | 323 (14.5) | 17 (5.7) | 261 (11.0) |
|  | Higher education | 10 (4.0) | 92 (4.1) | 12 (4.0) | 71 (3.0) |
| **Socioeconomic status** | Poorest | 55 (22.0) | 385 (17.2) | 84 (28.1) | 571 (24.0) |
|  | Poor | 54 (21.6) | 429 (19.2) | 66 (22.1) | 498 (20.9) |
|  | Median | 42 (16.8) | 423 (18.9) | 53 (17.7) | 442 (18.6) |
|  | Less poor | 46 (18.4) | 478 (21.4) | 62 (20.7) | 447 (18.8) |
|  | Least poor | 53 (21.2) | 519 (23.2) | 34 (11.4) | 424 (17.8) |
| **Household size** | 1-5 | 90 (36.0) | 607 (27.2) | 88 (29.6) | 615 (26.0) |
|  | 6-9 | 118 (47.2) | 1326 (59.5) | 171 (57.6) | 1409 (59.5) |
|  | 10-31 | 42 (16.8) | 296 (13.3) | 38 (12.8) | 344 (14.5) |
| Study endpoints-baseline |  | Class 1 N=149  Class 5 N=116 | Class 1 N=1073  Class 5 N=1185 | Class 1 N=153  Class 5 N=159 | Class 1 N=1164  Class 5 N=1234 |
| **Anaemia prevalence** | Age-sex specific | 93 (42.9) | 980 (45.5) | 98 (45.2) | 1016 (45.5) |
|  | Severe (<70g/L) | 1 (0.5) | 13 (0.6) | 1 (0.5) | 13 (0.6) |
|  | Moderate (70-89 g/L) | 8 (3.7) | 35 (1.6) | 9 (4.1) | 46 (2.1) |
|  | Mild (90-109 g/L) | 43 (19.8) | 487 (22.6) | 44 (20.3) | 474 (21.2) |
|  | None (≥110 g/L) | 165 (76.0) | 1621 (75.2) | 163 (75.1) | 1701 (76.1) |
| **Haemoglobin (g/L)** | Mean (sd) | 116.6 (14.1) | 117.4 (12.9) | 117.5 (15.0) | 117.5 (13.6) |
| ***P.falciparum* prevalence** ^b^ |  | - - | - - | 19 (9.1) | 292 (13.3) |
| **Class 1** ^c^ |  |  |  |  |  |
| Score: 0-20 | Sustained attention ^d^ | 11.0 (6.8) [0, 20] | 12.0 (6.6) [0, 20] | 12.3 (6.7) [0, 20] | 12.1 (6.6) [0, 20] |
| Score: 0-20 | Spelling | 8.2 (4.3) [0, 19] | 8.6 (4.5) [0, 20] | 7.1 (4.2) [0, 18] | 7.7 (4.4) [0, 20] |
| Score: 0-30 | Arithmetic | 2.8 (2.8) [0, 13] | 2.5 (2.3) [0, 17] | 2.8 (2.9) [0, 13] | 2.5 (2.4) [0, 15] |
| **Class 5**  ^c^ |  |  |  |  |  |
| Score: 0-20 | Sustained attention ^d^ | 9.8 (5.8) [0, 20] | 9.9 (6.0) [0, 20] | 9.5 (5.8) [0, 20] | 10.6 (5.6) [0, 20] |
| Score: 0-78 | Spelling | 24.6 (11.1) [2, 52] | 28.2 (11.8) [0, 63] | 25.1 (11.2) [1, 51] | 25.9 (11.2) [1, 59] |
| Score: 0-38 | Arithmetic | 28.3 (6.6) [5, 38] | 29.5 (5.5) [0, 38] | 27.8 (7.2) [3, 38] | 28.6 (5.6) [0, 38] |

^a^ % of non-missing children in each study group presented for categorised data, where data is continuous mean(sd) is presented.

^b^ Not measured at baseline in the control group;

^c^ Presented as mean(sd) [min,max]

^d^ In class 1 sustained attention was measured by the “pencil tap test” and in class 5 sustained attention was measured by the “two digit code transmission test”.
