# Supplementary material for: Impact of Intermittent Screening and Treatment for Malaria among School Children in Kenya: A Cluster Randomised Trial
Source: PLoS Med. 2014 Jan 28;11(1):e1001594. doi: 10.1371/journal.pmed.1001594 (PMC3904819; doi:10.1371/journal.pmed.1001594)
Supplement: Table S5 — Baseline measures for study children with missing 24-months follow-up education data versus those not missing 24-months follow-up education data across both the control and intervention groups. (DOC) [file pmed.1001594.s010.doc]

**Table S5. Baseline measures for study children with missing 24 months follow-up education data vs. those not missing 24 months follow-up education data across both the control and intervention groups.**

| Characteristic; n (%) ^a^ |  | **CONTROL GROUP** | | **INTERVENTION GROUP** | |
| --- | --- | --- | --- | --- | --- |
|  |  | Missing outcome data | Outcome data available | Missing outcome data | Outcome data available |
| Child characteristics |  | N=543 | N=1980 | N=584 | N=2126 |
| **Age** | Mean (sd) | 10.5 (3.1) | 10.0 (2.8) | 10.9 (3.1) | 10.2 (2.7) |
|  | 5-9 | 213 (39.2) | 828 (41.8) | 202 (34.6) | 867 (40.8) |
|  | 10-12 | 161 (29.7) | 716 (36.2) | 167 (28.6) | 758 (35.7) |
|  | 13-20 | 169 (31.1) | 436 (22.0) | 215 (36.8) | 501 (23.6) |
| **Sex** | Male | 271 (49.9) | 986 (49.8) | 270 (46.2) | 1049 (49.3) |
| **Child sleeps under net** | Usually | 343 (65.2) | 1325 (67.8) | 345 (61.0) | 1337 (63.7) |
|  | Last night | 334 (97.4) | 1272 (96.0) | 328 (95.1) | 1281 (95.8) |
| **Nutritional Status** | Underweight | 49 (26.1) | 217 (27.2) | 37 (22.8) | 194 (24.1) |
|  | Stunted | 114 (23.7) | 486 (25.5) | 121 (25.0) | 491 (24.8) |
|  | Thin | 90 (18.7) | 392 (20.6) | 74 (15.3) | 376 (19.0) |
| Household characteristics |  |  |  |  |  |
| **Parental Education** | No schooling | 167 (31.8) | 559 (28.7) | 229 (40.4) | 696 (33.1) |
|  | Primary schooling | 258 (49.1) | 1034 (53.1) | 271 (47.8) | 1110 (52.9) |
|  | Secondary schooling | 82 (15.6) | 271 (13.9) | 46 (8.1) | 232 (11.0) |
|  | Higher education | 18 (3.4) | 84 (4.3) | 21 (3.7) | 62 (3.0) |
| **Socioeconomic status** | Poorest | 102 (19.4) | 338 (17.3) | 138 (24.3) | 517 (24.5) |
|  | Poor | 119 (22.6) | 364 (18.6) | 125 (22.0) | 439 (20.8) |
|  | Median | 92 (17.5) | 373 (19.1) | 110 (19.3) | 385 (18.2) |
|  | Less poor | 86 (16.3) | 438 (22.4) | 109 (19.2) | 400 (18.9) |
|  | Least poor | 128 (24.3) | 444 (22.7) | 87 (15.3) | 371 (17.6) |
| **Household size** | 1-5 | 163 (31.0) | 534 (27.3) | 152 (26.9) | 551 (26.3) |
|  | 6-9 | 293 (55.7) | 1151 (58.9) | 335 (59.2) | 1245 (59.3) |
|  | 10-31 | 70 (13.3) | 268 (13.7) | 79 (14.0) | 303 (14.4) |
| Study endpoints-baseline |  | Class 1 N=259  Class 5 N=284 | Class 1 N=963  Class 5 N=1017 | Class 1 N=253  Class 5 N=331 | Class 1 N=1064  Class 5 N=1062 |
| **Anaemia prevalence** | Age-sex specific | 213 (44.9) | 860 (45.3) | 211 (44.8) | 903 (45.6) |
|  | Severe (<70g/L) | 2 (0.4) | 12 (0.6) | 1 (0.2) | 13 (0.7) |
|  | Moderate (70-89 g/L) | 10 (2.1) | 33 (1.7) | 9 (1.9) | 46 (2.3) |
|  | Mild (90-109 g/L) | 104 (21.9) | 426 (22.4) | 91 (19.3) | 427 (21.6) |
|  | None (≥110 g/L) | 358 (75.5) | 1428 (75.2) | 370 (78.6) | 1494 (75.5) |
| **Haemoglobin (g/L)** | Mean (sd) | 117.4 (13.4) | 117.3 (12.9) | 118.7 (13.6) | 117.2 (13.7) |
| ***P.falciparum* prevalence** ^b^ |  | - - | - | 47 (10.2) | 264 (13.6) |
| **Class 1** ^c^ |  |  |  |  |  |
| Score: 0-20 | Sustained attention ^d^ | 11.8 (6.6) [0, 20] | 11.9 (6.7) [0, 20] | 11.9 (6.6) [0, 20] | 12.2 (6.6) [0, 20] |
| Score: 0-20 | Spelling | 8.5 (4.2) [0, 19] | 8.6 (4.6) [0, 19] | 7.6 (4.6) [0, 19] | 7.7 (4.4) [0, 20] |
| Score: 0-30 | Arithmetic | 2.5 (2.3) [0, 12] | 2.6 (2.4) [0, 17] | 2.6 (2.7) [0, 13] | 2.6 (2.4) [0, 15] |
| **Class 5**  ^c^ |  |  |  |  |  |
| Score: 0-20 | Sustained attention ^d^ | 9.9 (6.1) [0, 20] | 9.9 (6.0) [0, 20] | 9.6 (5.6) [0, 20] | 10.7 (5.7) [0, 20] |
| Score: 0-78 | Spelling | 25.4 (11.6) [0, 53] | 28.6 (11.7) [0, 63] | 23.1 (11.1) [1, 59] | 26.6 (11.1) [1, 59] |
| Score: 0-38 | Arithmetic | 28.7 (6.3) [4, 38] | 29.5 (5.3) [0, 38] | 27.7 (6.3) [3, 38] | 28.8 (5.6) [0, 38] |

^a^ % of non-missing children in each study group presented for categorised data, where data is continuous mean(sd) is presented.

^b^ Not measured at baseline in the control group;

^c^ Presented as mean(sd) [min,max]

^d^ In class 1 sustained attention was measured by the “pencil tap test” and in class 5 sustained attention was measured by the “two digit code transmission test”.
