# Supplementary material for: Impact of Intermittent Screening and Treatment for Malaria among School Children in Kenya: A Cluster Randomised Trial
Source: PLoS Med. 2014 Jan 28;11(1):e1001594. doi: 10.1371/journal.pmed.1001594 (PMC3904819; doi:10.1371/journal.pmed.1001594)
Supplement: Table S6 — Results from missing data analysis for sustained attention. Effect of the IST intervention at 9- and 24-months follow-up on sustained attention outcomes for younger (class 1) and older (class 5) children combined using a longitudinal, random effects regression modeling approach. Results presented (i) for all children with either 9- or 24-months follow-up measurements of the outcome (unadjusted), (ii) for those with baseline measurements of the outcome and accounting for age, sex, and stratification effects as the primary pre-specified analysis, and (iii) for those additionally with baseline measures of parental education, SES, and baseline educational level (measured by baseline spelling) as further predictors of missingness. (DOC) [file pmed.1001594.s011.doc]

**Table S6. Results from missing data analysis for sustained attention.** Effect of the IST intervention at 9 and 24 months follow-up on sustained attention outcomes for younger (class 1) and older (class 5) children combined using a longitudinal, random effects regression modeling approach. Results presented (i) for all children with either 9 or 24 months follow-up measurements of the outcome (unadjusted), (ii) for those with baseline measurements of the outcome and accounting for age, sex and stratification effects as the primary pre-specified analysis, and (iii) for those additionally with baseline measures of parental education, SES and baseline educational level (measured by baseline spelling) as further predictors of missingness.

| **Sustained attention (score: 0-20)** | **Control**  **(50 schools)** | | **Intervention**  **(51 schools)** | | **Mean difference ^d^**  **(95% CI)** | **p-value** ^e^ | **ICC (95% CI)** | |
| --- | --- | --- | --- | --- | --- | --- | --- | --- |
|  |  |  |  |  |  |  | School | Child |
| **CLASS 1 ^b^** |  | **Mean (SD) ^a^** |  | **Mean (SD) ^a^** |  |  |  |  |
| **Unadjusted** |  |  |  |  |  |  |  |  |
| 9-months | 1070 | 8.48 (3.63) | 1162 | 8.43 (3.76) | -0.05 (-0.53,0.44) | 0.409 | 0.04 (0.02,0.06) | 0.17 (0.13,0.22) |
| 24-months | 960 | 13.45 (5.15) | 1059 | 13.20 (4.96) | -0.25 (-0.75,0.24) |
| **Adjusted** |  |  |  |  |  |  |  |  |
| 9-months | 1030 | 8.52 (3.65) | 1144 | 8.43 (3.77) | -0.16 (-0.61,0.30) | 0.311 | 0.03 (0.02,0.05) | 0.13 (0.09,0.18) |
| 24-months | 923 | 13.49 (5.15) | 1041 | 13.18 (4.96) | -0.41 (-0.88,0.06) |
| **Adjusted for predictors of missingness** | | | |  |  |  |  |  |
| 9-months | 1013 | 8.54 (3.67) | 1118 | 8.43 (3.77) | -0.02 (-0.43,0.46) | 0.385 | 0.02 (0.01,0.04) | 0.12 (0.08,0.17) |
| 24-months | 908 | 13.49 (5.16) | 1017 | 13.19 (5.00) | -0.21 (-0.67,0.26) |
| **CLASS 5 ^c^** |  | **Mean (SD) ^a^** |  | **Mean (SD) ^a^** |  |  |  |  |
| **Unadjusted** |  |  |  |  |  |  |  |  |
| 9-months | 1180 | 13.38 (5.45) | 1231 | 13.35 (5.13) | -0.07 (-0.65,0.51) | 0.083 | 0.04 (0.03,0.07) | 0.52 (0.49,0.55) |
| 24-months | 1007 | 14.22 (4.90) | 1052 | 14.66 (5.13) | 0.31 (-0.29,0.91) |
| **Adjusted** |  |  |  |  |  |  |  |  |
| 9-months | 1178 | 13.38 (5.45) | 1221 | 13.40 (5.10) | -0.14 (-0.65,0.37) | 0.122 | 0.04 (0.03,0.07) | 0.40 (0.36,0.44) |
| 24-months | 1006 | 14.21 (4.90) | 1044 | 14.70 (5.10) | 0.19 (-0.33,0.72) |
| **Adjusted for predictors of missingness** | | |  |  |  |  |  |  |
| 9-months | 1141 | 13.39 (5.42) | 1203 | 13.40 (5.10) | -0.02 (-0.54,0.51) | 0.160 | 0.05 (0.03,0.07) | 0.37 (0.34,0.42) |
| 24-months | 971 | 14.24 (4.85) | 1028 | 14.69 (4.58) | 0.29 (-0.25,0.84) |

^a^ Mean score and sd at follow-up ^b^ Pencil tap test was conducted at baseline and single digit code transmission task was conducted at 9 and 24 months follow-ups.

^c^ Double digit code transmission was conducted at baseline and both follow up visits.

^d^ Mean difference (intervention-control) presented for continuous outcomes (scores on attention task) and are obtained from random effects regression analysis accounting for school-level clustering and repeated measures on children.

^e^ p-value for the comparison of the intervention effect at 12 months to 24 months

**Unadjusted**: All children with outcome measures, not adjusted for any baseline or study design characteristics.

**Adjusted**: for baseline age, sex, school mean exam score and literacy group (to account for stratification) and baseline measure of the outcome, where available.

**Adjusted for predictors of missingness**: for baseline age, sex, school mean exam score and literacy group (to account for stratification) and baseline measure of the outcome, where available. Additionally adjusted for parental education, SES and baseline educational level as measured by baseline spelling score (standardized by subtracting year-group baseline mean and scaled by year-group sd).
