# Supplementary material for: Impact of Intermittent Screening and Treatment for Malaria among School Children in Kenya: A Cluster Randomised Trial
Source: PLoS Med. 2014 Jan 28;11(1):e1001594. doi: 10.1371/journal.pmed.1001594 (PMC3904819; doi:10.1371/journal.pmed.1001594)
Supplement: Table S7 — Results from missing data analysis for spelling. Effect of the IST intervention at 9- and 24-months follow-up on spelling outcomes for younger (class 1) and older (class 5) children combined using a longitudinal, random effects regression modeling approach. Results presented (i) for all children with either 9- or 24-months follow-up measurements of the outcome (unadjusted), (ii) for those with baseline measurements of the outcome and accounting for age, sex, and stratification effects as the primary pre-specified analysis, and (iii) for those additionally with baseline measures of parental education, SES, and baseline educational level (measured by baseline spelling) as further predictors of missingness. (DOC) [file pmed.1001594.s012.doc]

**Table S7: Results from missing data analysis for spelling.** Effect of the IST intervention at 9 and 24 months follow-up on spelling outcomes for younger (class 1) and older (class 5) children combined using a longitudinal, random effects regression modeling approach. Results presented (i) for all children with either 9 or 24 months follow-up measurements of the outcome (unadjusted), (ii) for those with baseline measurements of the outcome and accounting for age, sex and stratification effects as the primary pre-specified analysis, and (iii) for those additionally with baseline measures of parental education, SES and baseline educational level (measured by baseline spelling) as further predictors of missingness.

| **Spelling score** | **Control**  **(50 schools)** | | **Intervention**  **(51 schools)** | | **Mean difference ^d^**  **(95% CI)** | **p-value** ^e^ | **ICC (95% CI)** | |
| --- | --- | --- | --- | --- | --- | --- | --- | --- |
|  |  |  |  |  |  |  | School | Child |
| **CLASS 1 ^b^** |  | **Mean (SD) ^a^** |  | **Mean (SD) ^a^** |  |  |  |  |
| **Unadjusted** |  |  |  |  |  |  |  |  |
| 9-months | 1068 | 11.70 (4.59) | 1162 | 10.47 (4.57) | -1.24 (-2.00,-0.48) | 0.094 | 0.20 (0.15,0.25) | 0.61 (0.58,0.65) |
| 24-months | 961 | 12.03 (3.05) | 1062 | 11.04 (3.49) | -0.98 (-1.74,-0.21) |
| **Adjusted** |  |  |  |  |  |  |  |  |
| 9-months | 1060 | 11.69 (4.59) | 1133 | 10.49 (4.58) | -0.79 (-1.28,-0.30) | 0.116 | 0.10 (0.07,0.14) | 0.43 (0.39,0.47) |
| 24-months | 954 | 12.02 (3.05) | 1036 | 11.04 (3.50) | -0.54 (-1.04,-0.05) |
| **Adjusted for predictors of missingness** | | |  |  |  |  |  |  |
| 9-months | 1049 | 11.70 (4.59) | 1121 | 10.49 (4.58) | -0.75 (-1.23,-0.27) | 0.178 | 0.09 (0.07,0.14) | 0.42 (0.38,0.46) |
| 24-months | 944 | 12.05 (3.03) | 1025 | 11.03 (3.50) | -0.54 (-1.02,-0.05) |
| **CLASS 5 ^c^** |  | **Mean (SD) ^a^** |  | **Mean (SD) ^a^** |  |  |  |  |
| **Unadjusted** |  |  |  |  |  |  |  |  |
| 9-months | 1169 | 31.34 (12.61) | 1223 | 28.73 (12.36) | -2.69 (-5.10,-0.27) | 0.001 | 0.21 (0.16,0.26) | 0.85 (0.84,0.87) |
| 24-months | 1010 | 35.28 (12.91) | 1060 | 33.97 (12.79) | -1.70 (-4.13,0.73) |
| **Adjusted** |  |  |  |  |  |  |  |  |
| 9-months | 1154 | 31.37 (12.60) | 1214 | 28.76 (12.34) | -0.28 (-1.16,0.60) | 0.001 | 0.08 (0.06,0.12) | 0.43 (0.40,0.47) |
| 24-months | 996 | 35.33 (12.85) | 1052 | 34.04 (12.75) | 0.68 (0.22,1.58) |
| **Adjusted for predictors of missingness** | | |  |  |  |  |  |  |
| 9-months | 1131 | 31.49 (12.69) | 1198 | 28.69 (12.36) | -0.18 (-1.07,0.70) | 0.003 | 0.08 (0.06,0.12) | 0.43 (0.40,0.47) |
| 24-months | 974 | 35.57 (12.81) | 1037 | 33.98 (12.77) | 0.73 (-0.18,1.63) |

^a^ Mean score and sd at follow-up based on the data

**^b^** The same class 1 spelling task was given at baseline, 9 and 24 months follow-ups, with different words used for the 24 month follow-up and was scored 0-20.

**^c^** The same class 5 spelling task was given at baseline, 9 and 24 months follow-ups, with different words used for the 24 month follow-up and was scored 0-78.

^d^ Mean difference (intervention-control) presented for continuous outcomes (scores on spelling task) and are obtained from random effects regression analysis accounting for school-level clustering and repeated measures on children.

^e^ p-value for the comparison of the intervention effect at 12 months to 24 months

**Unadjusted**: All children with outcome measures, not adjusted for any baseline or study design characteristics.

**Adjusted**: for baseline age, sex, school mean exam score and literacy group (to account for stratification) and baseline measure of the outcome, where available.

**Adjusted for predictors of missingness**: for baseline age, sex, school mean exam score and literacy group (to account for stratification) and baseline measure of the outcome, where available. Additionally adjusted for parental education, SES and baseline educational level as measured by baseline spelling score (standardized by subtracting year-group baseline mean and scaled by year-group sd).
