# Supplementary material for: Impact of Intermittent Screening and Treatment for Malaria among School Children in Kenya: A Cluster Randomised Trial
Source: PLoS Med. 2014 Jan 28;11(1):e1001594. doi: 10.1371/journal.pmed.1001594 (PMC3904819; doi:10.1371/journal.pmed.1001594)
Supplement: Table S8 — Sensitivity analyses considering transfers across the study period. Effect of the IST intervention at 12- and 24-months follow-up on health outcomes for study children. Results presented (i) for all children with either 12- or 24-months follow-up measurements of the outcome (unadjusted) with children who transferred schools excluded and (ii) for those with baseline measurements of each outcome and accounting for age, sex, and stratification effects as the primary pre-specified analysis with children who transferred schools excluded. (DOC) [file pmed.1001594.s013.doc]

**Table S8. Sensitivity analyses considering transfers across the study period.** Effect of the IST intervention at 12 and 24 months follow-up on health outcomes for study children. Results presented (i) for all children with either 12 or 24 months follow-up measurements of the outcome (unadjusted) with children who transferred schools excluded and (ii) for those with baseline measurements of each outcome and accounting for age, sex and stratification effects as the primary pre-specified analysis with children who transferred schools excluded.

| **Outcome** | **Control**  **(50 schools)** | | **Intervention**  **(51 schools)** | | **Risk ratio^b^**  **(95% CI)** | **p-value** | **Cluster-size; range (average)** |
| --- | --- | --- | --- | --- | --- | --- | --- |
|  |  | **n (%)^a^** |  | **n (%)^a^** |  |  |  |
| **12 month follow-up** | N=2439 |  | N=2574 |  |  |  |  |
| **Prevalence of anaemia^c^** |  |  |  |  |  |  |  |
| Unadjusted | 2117 | 827 (39.1%) | 2255 | 906 (40.2%) | 1.03 (0.91,1.16) | 0.640 | 15-54 (43.3) |
| Adjusted | 2023 | 780 (38.6%) | 2106 | 847 (40.2%) | 1.02 (0.93,1.13) | 0.670 | 15-54 (40.9) |
| **Prevalence of *P.falciparum*** |  |  |  |  |  |  |  |
| Unadjusted | 2078 | 300 (14.4%) | 2235 | 243 (10.9%) | 0.76 (0.49,1.19) | 0.234 | 11-54 (42.7) |
| Adjusted ^d^ | 2078 | 300 (14.4%) | 2235 | 243 (10.9%) | 0.72 (0.46,1.11) | 0.139 | 11-54 (42.7) |
| **24 months follow-up** | N=2362 |  | N=2417 |  |  |  |  |
| **Prevalence of anaemia^c^** |  |  |  |  |  |  |  |
| Unadjusted | 1929 | 770 (39.9%) | 1999 | 843 (42.2%) | 1.06 (0.91,1.22) | 0.463 | 15-52 (39.3) |
| Adjusted | 1845 | 728 (39.5%) | 1862 | 780 (41.9%) | 1.01 (0.90,1.12) | 0.920 | 14-52 (37.1) |
| **Prevalence of *P.falciparum*** |  |  |  |  |  |  |  |
| Unadjusted | 1908 | 162 (8.5%) | 1972 | 239 (12.2%) | 1.42 (0.83,2.43) | 0.206 | 15-52 (38.8) |
| Adjusted ^d^ | 1908 | 162 (8.5%) | 1972 | 239 (12.2%) | 1.49 (0.86,2.57) | 0.154 | 15-52 (38.8) |

N=number of children eligible for follow up (not withdrawn or deceased)

**^a^** Number and percentage with outcome

**^b^** Risk ratios presented for binary outcomes (anaemia & *P. falciparum* prevalence) and are obtained from GEE analysis accounting for school-level clustering.

**^c^** Age-sex specific anaemia was defined using age and sex corrected WHO thresholds of haemoglobin concentration: <110g/l in children under 5 years; <115g/l in children 5 to 11 years; <120g/l in females 12 years and over and males 12 to 14.99 years old; and <130g/l in males ≥ 15 years. All female adolescents are assumed to not be pregnant

**^d^** Not including baseline *P.falciparum* prevalence

**Unadjusted**: All children with outcome measures, not adjusted for any baseline or study design characteristics.

**Adjusted**: for baseline age, sex, school mean exam score and literacy group (to account for stratification) and baseline measure of the outcome, where available
