# Supplementary material for: Impact of Intermittent Screening and Treatment for Malaria among School Children in Kenya: A Cluster Randomised Trial
Source: PLoS Med. 2014 Jan 28;11(1):e1001594. doi: 10.1371/journal.pmed.1001594 (PMC3904819; doi:10.1371/journal.pmed.1001594)
Supplement: Table S9 — Analysis stratified by categories of P. falciparum prevalence at baseline. Effect of the IST intervention at 12- and 24-months follow-up on the prevalence of anaemia, by baseline prevalence category of P. falciparum (control school prevalence estimated using 12-month follow-up data) with adjustment for age, sex, and stratification effects. (DOC) [file pmed.1001594.s014.doc]

**Table S9. Analysis stratified by categories of *P.falciparum* prevalence at baseline.** Effect of the IST intervention at 12 and 24 months follow-up on the prevalence of anaemia, by baseline prevalence category of *P.falciparum* (control school prevalence estimated using 12 month follow-up data) with adjustment for age, sex and stratification effects.

| **Prevalence of anaemia** | **Control**  **(50 schools)** | | **Intervention**  **(51 schools)** | | **Risk ratio^c^**  **(95% CI)** | **p-value** |
| --- | --- | --- | --- | --- | --- | --- |
|  |  | **n (%)^b^** |  | **n (%)^b^** |  |  |
| **Follow-up 12 months** | N=2478 |  | N=2631 |  |  |  |
| **Baseline % *P.falciparum* ^a^** |  |  |  |  |  |  |
| <5% | 787 | 265 (33.7%) | 751 | 270 (36.0%) | 1.01 (0.84,1.23) | 0.578 |
| 5-19.9% | 606 | 220 (36.3%) | 858 | 358 (41.7%) | 1.09 (0.95,1.26) |
| ≥20% | 655 | 303 (46.3%) | 533 | 230 (43.2%) | 0.99 (0.87,1.13) |
| **Follow-up 24 months** | N=2468 |  | N=2619 |  |  |  |
| **Baseline % *P.falciparum* ^a^** |  |  |  |  |  |  |
| <5% | 740 | 264 (35.7%) | 710 | 243 (34.2%) | 0.95 (0.78,1.16) | 0.840 |
| 5-19.9% | 572 | 226 (39.5%) | 803 | 364 (45.3%) | 0.99 (0.86,1.14) |
| ≥20% | 623 | 275 (44.1%) | 514 | 235 (45.7%) | 1.03 (0.86,1.24) |

N=numbers not withdrawn or died by the time of follow-up.

^a^ Control school *P.falciparum* prevalence was estimated using 12 month follow-up data.

**^b^** Number and percentage with outcome

^c^ Risk ratios presented are obtained from GEE analysis accounting for school-level clustering and baseline outcome (anaemia).
