# Supplementary material for: Impact of Intermittent Screening and Treatment for Malaria among School Children in Kenya: A Cluster Randomised Trial
Source: PLoS Med. 2014 Jan 28;11(1):e1001594. doi: 10.1371/journal.pmed.1001594 (PMC3904819; doi:10.1371/journal.pmed.1001594)
Supplement: Table S10 — Analysis stratified by number of AL treatments received. Effect of the IST intervention at 12- and 24-months follow-up within the IST intervention group by number of positive results and subsequent treatments received at the individual level. (DOC) [file pmed.1001594.s015.doc]

**Table S10. Analysis stratified by number of AL treatments received.** Effect of the IST intervention at 12 and 24 months follow-up within the IST intervention group by number of positive results and subsequent treatments received at the individual level.

| **Prevalence of anaemia** | **Intervention**  **(51 schools)** | | **Risk ratio^d^**  **(95% CI)** | **p-value** |
| --- | --- | --- | --- | --- |
| **Follow-up 12 months** | **N ^a^** | **n (%)^b^** |  |  |
| **No. treatments received ^c^** | N=2293 |  |  |  |
| **0** | 1417 | 545 (38.5%) | 0 | 0.839 |
| **1** | 588 | 241 (41.0%) | 0.99 (0.90, 1.09) |
| **2-3** | 288 | 131 (45.5%) | 1.04 (0.91, 1.19) |
| **Follow-up 24 months** |  |  |  |  |
| **No. treatments received ^c^** | N=2169 |  |  |  |
| **0** | 1336 | 546 (40.9%) | 0 | 0.470 |
| **1-2** | 563 | 233 (41.4%) | 0.96 (0.88, 1.05) |
| **3-5** | 270 | 129 (47.8%) | 1.03 (0.88, 1.21) |

**^a^** Number of children receiving by the number of visits at which they required and received treatment for *Plasmodium* infection

**^b^** Number and percentage of children with anaemia at follow-up

^c^ At 12 month follow-up the maximum possible treatments was three as three IST rounds had been completed. By the 24 month follow-up the maximum possible treatments was five as five IST rounds had been completed

^d^ Risk ratios presented are obtained from GEE analysis accounting for school-level clustering and baseline outcome (anaemia).
