# Supplementary material for: Impact of Intermittent Screening and Treatment for Malaria among School Children in Kenya: A Cluster Randomised Trial
Source: PLoS Med. 2014 Jan 28;11(1):e1001594. doi: 10.1371/journal.pmed.1001594 (PMC3904819; doi:10.1371/journal.pmed.1001594)
Supplement: Text S1 — Methods for the missing data models. (DOC) [file pmed.1001594.s016.doc]

**Text S1: Methods for the missing data models**

We performed a missing data analysis for the primary outcomes of age-specific anaemia and sustained attention and for the secondary outcome of spelling, for which we identified a negative effect of the IST intervention in Class 1 children. In order to gain power and account for missing data, we used a likelihood-based approach and fitted random effects models to the one-year and two-year follow-up data simultaneously. We included the following variables in all models: age, sex, literacy group, school-mean exam score and a baseline measure, where possible. The logit link was used for binary outcomes to obtain odds ratios of the intervention effect. As a consequence, the intervention effects from these models are not directly comparable to the population-averaged risk ratios obtained from the GEE model. As a consequence, we also present results from the unadjusted models for comparison to the models additionally adjusted for predictors of missingness. Time was modeled as a categorical variable in all models so that we did not assume a specific linear effect of time. Specifically, we allowed the IST effect to differ at the two time-points by including an interaction between IST and time. We additionally adjusted for variables seen to predict missingness. See Tables S1 and S2 for predictors of missingness for health outcomes at follow up 1 and follow-up 2 respectively, in both the control and intervention groups. Tables S3and S4 show the equivalent data for educational outcomes at follow up 1 and follow-up 2 respectively. Specifically we adjusted for SES, parental education and baseline educational level as measured by baseline spelling score (standardized by subtracting year-group baseline mean and scaled by year-group SD) in both the health and educational outcome analyses. By accounting for predictors of missingness in the models, we can obtain valid estimates of the intervention effect in the presence of missing follow-up data. Furthermore, we gain power by simultaneously modeling the follow up 1 and follow-up 2 data. For results of missingness analyses for health and education outcomes please see Tables S5 and S6.
